# Counterintuitive production of tumor-suppressive secretomes from Oct4- and c-Myc-overexpressing tumor cells and MSCs

**DOI:** 10.7150/thno.70549

**Published:** 2022-03-28

**Authors:** Kexin Li, Xun Sun, Rongrong Zha, Shengzhi Liu, Yan Feng, Tomonori Sano, Uma K. Aryal, Akihiro Sudo, Bai-Yan Li, Hiroki Yokota

**Affiliations:** 1 Department of Pharmacology, School of Pharmacy, Harbin Medical University, Harbin 150081, China.; 2 Department of Biomedical Engineering, Indiana University Purdue University Indianapolis, Indianapolis, IN 46202, USA.; 3 Department of Orthopedic Surgery, Mie University, Mie 514, Japan.; 4 Department of Comparative Pathobiology, Purdue University, West Lafayette, IN 47907, USA.; 5 Indiana Center for Musculoskeletal Health, Indiana University School of Medicine, Indianapolis, IN 46202, USA.; 6 Simon Cancer Center, Indiana University School of Medicine, Indianapolis, IN 46202, USA.

**Keywords:** breast cancer, cell competition, Oct4, c-Myc, enolase 1

## Abstract

**Background:** Advanced breast cancer frequently metastasizes to bone, but inhibiting tumor progression in chemotherapy may occasionally enhance tumorigenesis. Here, we employed a counterintuitive approach of overexpressing Yamanaka factors (Oct4, c-Myc, Sox2, and Klf4) and examined a conditioned medium (CM)-based treatment option with induced tumor-suppressing cells (iTSCs).

**Methods:** *In vitro* proliferation and migration assays were conducted using tumor cell lines derived from breast cancer, as well as prostate and pancreatic cancers, and osteosarcoma. The tumor-suppressing capability of iTSC-derived CM was evaluated using freshly isolated breast cancer tissues and a mouse model of mammary tumors and tumor-induced osteolysis. The regulatory mechanism was evaluated using Western blotting, immunoprecipitation, pull-down, gene overexpression, and RNA interference based on mass spectrometry-based proteomics data.

**Results:** The overexpression of Oct4 and c-Myc in tumor cells and MSCs, but not Sox2 or Klf4, generated anti-tumor CM, which suppressed the progression of mammary tumors and tumor-induced bone loss. Notably, CM downregulated histone demethylase, and PDL-1, a blocker of T-cell-based immune responses. Whole-genome proteomics predicted enolase 1 (Eno1), Hsp90ab1, Eef2, and vinculin as extracellular tumor suppressors. Specifically, CD44 was co-immunoprecipitated with Eno1 and the silencing of CD44 suppressed Eno1's anti-tumor action. The overexpression of Oct4 and c-Myc also generated secretomes that inhibited the development of bone-resorbing osteoclasts.

**Conclusions:** In analogous to cell competition in which Myc-overexpressing cells in Drosophila and mouse embryos remove neighboring cells with a lower level of Myc, this study presented the possibility of eliminating tumor cells by the secretory proteomes derived from Myc/Oc4-overexpressing iTSCs.

## Introduction

Eliminating tumor cells without using toxic agents is one of the ideal goals of cancer treatments. In Drosophila development, cell competition has been reported to play a unique role in regulating sequential growth and death of mosaic cells 1. According to the emerging mechanism, cells with higher protein synthesis eliminate neighboring cells with lower protein synthesis. This mechanism suggests the possibility of letting highly metabolically active cells remove less active cancer cells. While uncontrolled proliferation is a hallmark of tumor cells, many lines of evidence demonstrate that tumor growth is profoundly affected by the interactions with neighboring tumor and non-tumor cells 2. Instead of employing the chemotherapeutic inhibition of oncogenic signaling, an intriguing question is whether the activation of oncogenic signaling in tumor cells and non-tumor cells may elevate protein synthesis and produce tumor-suppressive secretomes.

We have previously shown that overexpressing Lrp5, β-catenin, Snail, or Akt converted many types of bone cells such as mesenchymal stem cells (MSCs), osteoblasts, osteocytes, and osteoclasts into induced tumor-suppressing (iTS) cells 3-7. iTS cells secrete well-known tumor-suppressing proteins such as p53 and Trail, as well as a group of uncommon tumor suppressors 3, including Hsp90ab1, calreticulin, and peptidylprolyl isomerase B. Most of these tumor suppressors exhibited a dichotomous role, in which they acted oncogenic in the cytoplasm of tumor cells and tumor-suppressive in the extracellular domain. The specific question in this study is any linkage of Yamanaka factors for generating induced pluripotent stem (iPS) cells with the formation of iTS cells.

iPS cells are engineered stem cells, which are produced through the dedifferentiation of adult somatic cells by overexpressing a specific set of transcription factors, c-Myc, Oct4, Sox2, and Klf4, named Yamanaka factors 8. Among those transcription factors, c-Myc is expressed constitutively in many cancer cells and activates tumorigenic genes that are involved in uncontrolled cell proliferation 9. It contributes to the oncogenesis of human cancers via Wnt/β-catenin, ERK/MAPK, and PI3K signaling pathways 10. Oct4 and Sox2 are oncogenes that are known to maintain the pluripotency and self-renewal of stem cells 11, 12. Wnt/β-catenin signaling regulates Oct4 and enhances pluripotency in stem and cancer cells 13, 14. Klf4 is involved in tumorigenesis, inflammation, DNA damage responses, and somatic cell reprogramming, and is related to varying diseases including cancers, vascular disorders, and cerebral malformations 15.

In the mosaic Myc-expressing mouse embryos, it is shown that cells with lower Myc levels are eliminated by apoptosis, and cells with higher Myc levels proliferate to fill the vacant spaces and become dominant 16. Drosophila studies also support the possibility of generating iTS cells by the overexpression of c-Myc. When a group of Drosophila cells expresses a higher level of dmyc, a homolog of c-Myc, than their neighbors, they outcompeted neighboring cells and even kill wild-type cells at a distance. Also, when dmyc-overexpressing cells are co-cultured with wild-type cells, the resulting conditioned medium (CM) is reported to induce cell death when incubated with wild-type cells. No data are available, however, regarding the outcome of overexpressing the other three Yamanaka factors.

In this study, we overexpressed each of the Yamanaka factors in tumor cells as well as non-tumor cells such as MSCs and T lymphocytes. Clinically, the use of patient-derived MSCs or T lymphocytes is advantageous over the application of tumor-derived iTS cells. Most notably, we discovered in this study that the effects of Oct4 and c-Myc were different from those of Sox2 and Klf4 and dependent on iTSC-generating cell types. The observed difference was linked to PI3K signaling, as well as the regulation of a lysine histone demethylase, Kdm3a, and PDL-1. Histone methylation is most commonly observed in the lysine residues of histone tails H3 and H4, and it regulates the activation and inactivation of chromatin. Kdm3a is reported to be overexpressed in multiple cancers, and epigenetically dysregulated methylation may serve as a tumor promoter 17. Also, blocking PDL-1 can prevent cancer cells from inactivating T cells through PD-1 18.

Whole-genome proteomics analysis revealed that a group of proteins, such as enolase 1 (Eno1), Hsp90ab1, Eef2, and vinculin, was enriched in Oct4- and c-Myc-overexpressing CMs. Interestingly, their extracellular forms acted as tumor suppressors, while their intracellular forms as tumor promoters. Furthermore, a cell adhesion molecule CD44, known to be involved in pro and anti-tumorigenic actions, was associated with the anti-tumor action of iTSC-derived CM 19. Taken together, this study demonstrated the unconventional role of Oct4 and c-Myc in suppressing tumor progression and indicated the complex role of cell competition among tumor and non-tumor cells. The result herein also sheds light on a century-old paradoxical observation 20, in which influential tumors may impede the progression of less-contentious tumors and their surgical removal could adversely affect the survival of patients.

## Results

### Tumor suppression *in vitro* by Oct4-overexpressing tumor cell-derived CM

**Figure 1:**
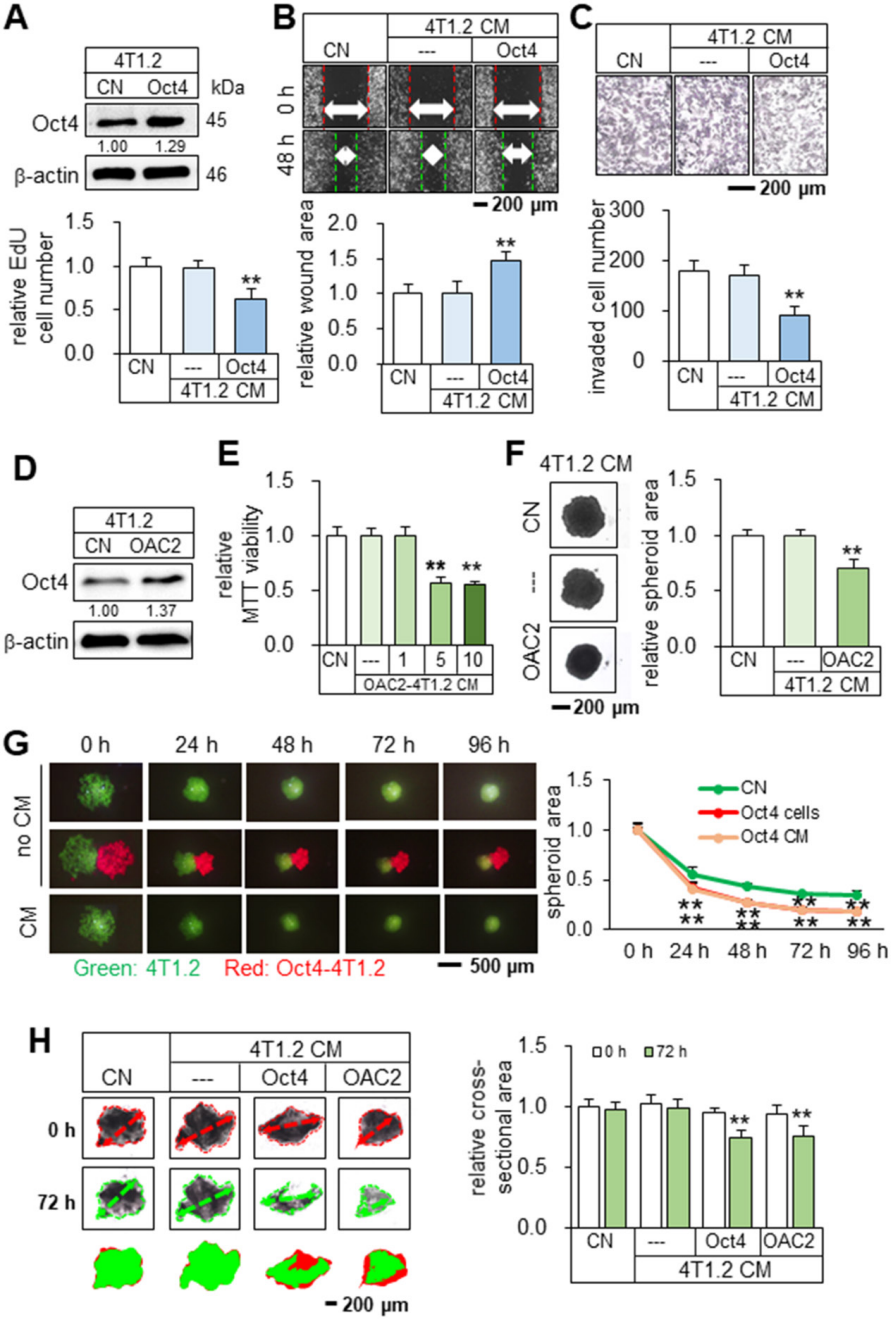
** Tumor suppression *in vitro* by Oct4-overexpressing CM derived from 4T1.2 mammary tumor cells.** The double asterisk indicates p < 0.01. CN = control, CM = conditioned medium, and Oct4 = Oct4 plasmids. (**A-C**) Reduction in EdU-based proliferation, scratch-based migration, and transwell invasion of parental 4T1.2 cells by Oct4-overexpressing CM that excluded exosomes. (**D**) Elevation of Oct4 in 4T1.2 cells by OAC2 treatment. (**E-F**) Reduction in MTT-based viability and tumor spheroids by OAC2 (5 µM)-treated 4T1.2-derived CM. (**G**) Time-dependent shrinkage of 4T1.2 tumor spheroids by Oct4-overexpressing tumor spheroids and their CM. (**H**) Reduction in *ex vivo* breast cancer tissue fragments by Oct4-overexpressing and OAC2-treated tumor cell-derived CM.

To test the tumor-suppressing effect of Oct4, CM from Oct4-overexpressing 4T1.2 mammary tumor cells was harvested after 24 h incubation and ultra-centrifuged to remove exosomes. We observed that Oct4-overexpressing CM (Oct4 CM) reduced the EdU-based proliferation in 2 days, scratch-based migration in 2 days, and transwell invasion of 4T1.2 parental cells in 2 days (Figure 1A-C). In contrast, Oct4-silenced CM reversed the responses and acted as a tumor promoter (Figure S1). We next employed OAC2, a pharmacological agent for activating Oct4. OAC2-treated 4T1.2-derived CM (OAC2 CM) also reduced the MTT-based viability of 4T1.2 parental cells and the growth of 4T1.2 tumor spheroids (Figure 1D-F). In the three-dimensional tumor spheroid assay, 4T1.2 tumor spheroids were shrunk in 1-4 days by Oct4-overexpressing tumor spheroids and their CM (Figure 1G). Furthermore, in the *ex vivo* breast cancer tissue assay, the size of cancer tissue fragments, freshly isolated from a patient with breast cancer, was significantly reduced in 3 days by the application of Oct4 CM and OAC2-treated CM (Figure 1H). The tumor-suppressing action of 4T1.2-derived OAC2 CM was observed not only for 4T1.2 parental cells but also for EO771 mammary tumor cells and MDA-MB-231 breast cancer cells (Figure S2). We hereafter employed CM after the ultracentrifugation.

### Tumor suppression and bone protection *in vivo* by Oct4 CM and OAC2 CM

**Figure 2:**
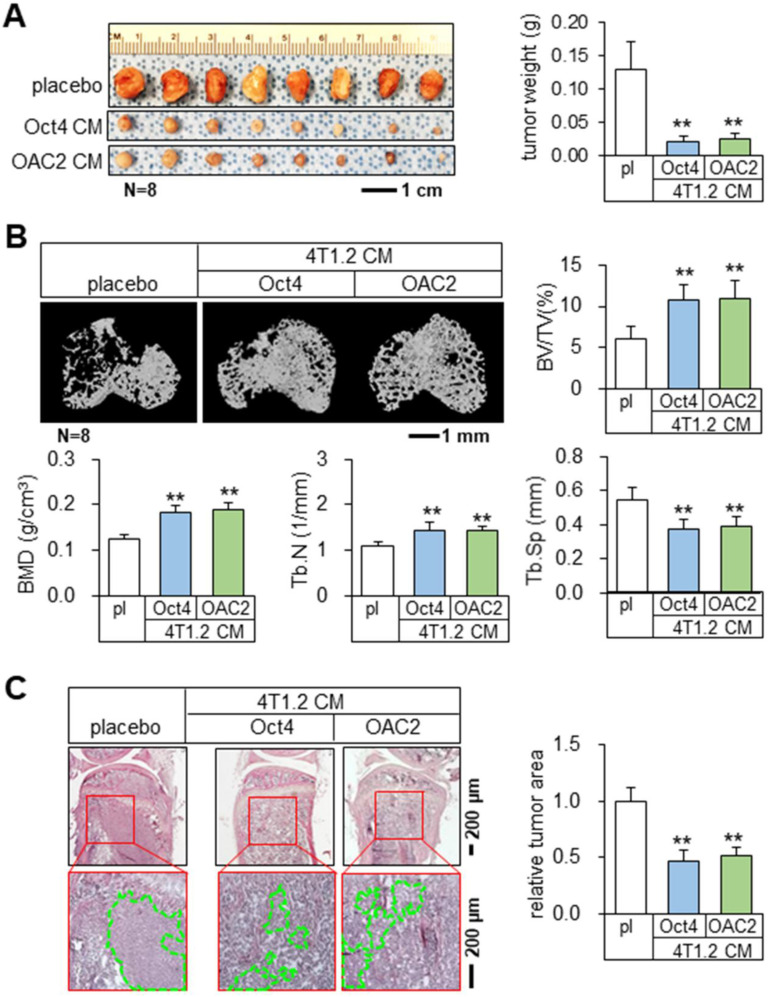
** Tumor suppression and bone protection *in vivo* by Oct4-overexpressing and OAC2-treated tumor cell-derived CMs.** The double asterisk indicates p < 0.01. pl = placebo, CM = conditioned medium, and Oct4 = Oct4 plasmids. (**A**) Significant reduction of mammary tumors by Oct4-overexpressing and OAC2-treated tumor cell-derived CMs. (**B**) Prevention of bone loss in the proximal tibia by Oct4-overexpressing and OAC2-treated tumor cell-derived CMs. BV/TV = bone volume ratio, BMD = bone mineral density, Tb.N = trabecular number, and Tb.Sp = trabecular separation. (**C**) Reduction in the tumor-invaded area by Oct4-overexpressing and OAC2-treated tumor cell-derived CMs.

Using the mouse models of mammary tumors and tibial osteolysis, we examined the effects of Oct4 and OAC2 in BALB/c mice, in which 50 μL of the concentrated Oct4 CM or OAC2 CM by 10-fold was administered daily as an intravenous injection. Notably, both Oct4 CM and OAC2 CM significantly reduced the growth of mammary tumors in 2 weeks (Figure 2A). Furthermore, these CMs prevented bone loss by elevating bone volume ratio, bone mineral density, and trabecular numbers, while decreasing trabecular separation that represented the spacing in trabecular bone (Figure 2B). Histological analysis with H&E-stained bone sections also supported the anti-tumor effect of CMs and the tumor-invaded areas were markedly reduced by Oct4 CM and OAC2 CM (Figure 2C). In contrast, the direct application of 1-10 μM OAC2 to 4T1.2 cells or the daily injection of OAC2 at 10 mg/kg to the mouse model of mammary tumors did not alter the proliferation of tumor cells or the progression of tumors (Figure S3A-B).

### Tumor suppression by c-Myc and Oct4 CMs

**Figure 3:**
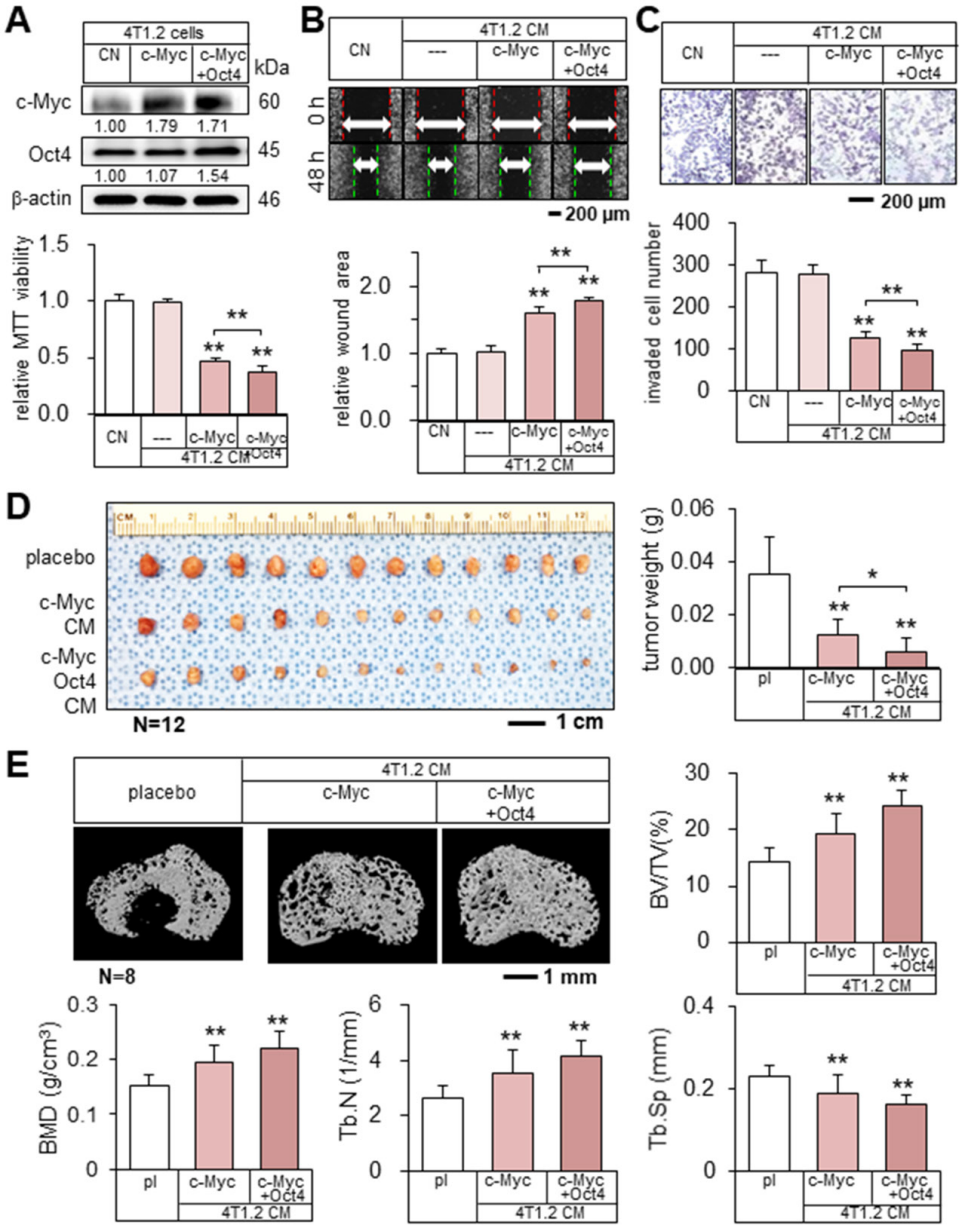
** Tumor suppression by c-Myc- and Oct4-overexpressing tumor cell-derived CMs.** The single and double asterisks indicate p < 0.05 and 0.01, respectively. CN = control, c-Myc = c-Myc plasmids, c-Myc + Oct4 = c-Myc and Oct4 plasmids, CM = conditioned medium, and pl = placebo. (**A-C**) Elevation of c-Myc and Oct4 in 4T1.2 cells, and the reduction in MTT-based viability, scratch-based migration, and transwell invasion by c-Myc- and Oct4-overexpressing tumor cell-derived CMs. (**D**) Significant reduction of mammary tumors in BALB/c mice by c-Myc- and Oct4-overexpressing tumor cell-derived CMs. (**E**) Prevention of bone loss in the proximal tibia of BALB/c mice by c-Myc- and Oct4-overexpressing tumor cell-derived CMs. BV/TV = bone volume ratio, BMD = bone mineral density, Tb.N = trabecular number, and Tb.Sp = trabecular separation.

Encouraged by the result with Oct4 CM, we examined the effect of c-Myc-overexpressing 4T1.2 tumor cell-derived CM (c-Myc CM). c-Myc CM also reduced MTT-based viability, scratch-based migration, and transwell invasion of parent tumor cells, and the simultaneous overexpression of c-Myc and Oct4 strengthened the inhibitory action (Figure 3A-C). Consistently, *in vivo* data in the BALB/c mouse models of mammary tumors and bone osteolysis supported the anti-tumor capability of c-Myc CM that significantly inhibited the growth of mammary tumors and the destruction of trabecular bone in the tibia (Figure 3D-E, Figure S3C). Moreover, the double overexpression of Oct4 and c-Myc (Oct4/c-Myc CM) enhanced inhibitory actions.

### Cancer cells and MSCs could become iTS cells

**Figure 4:**
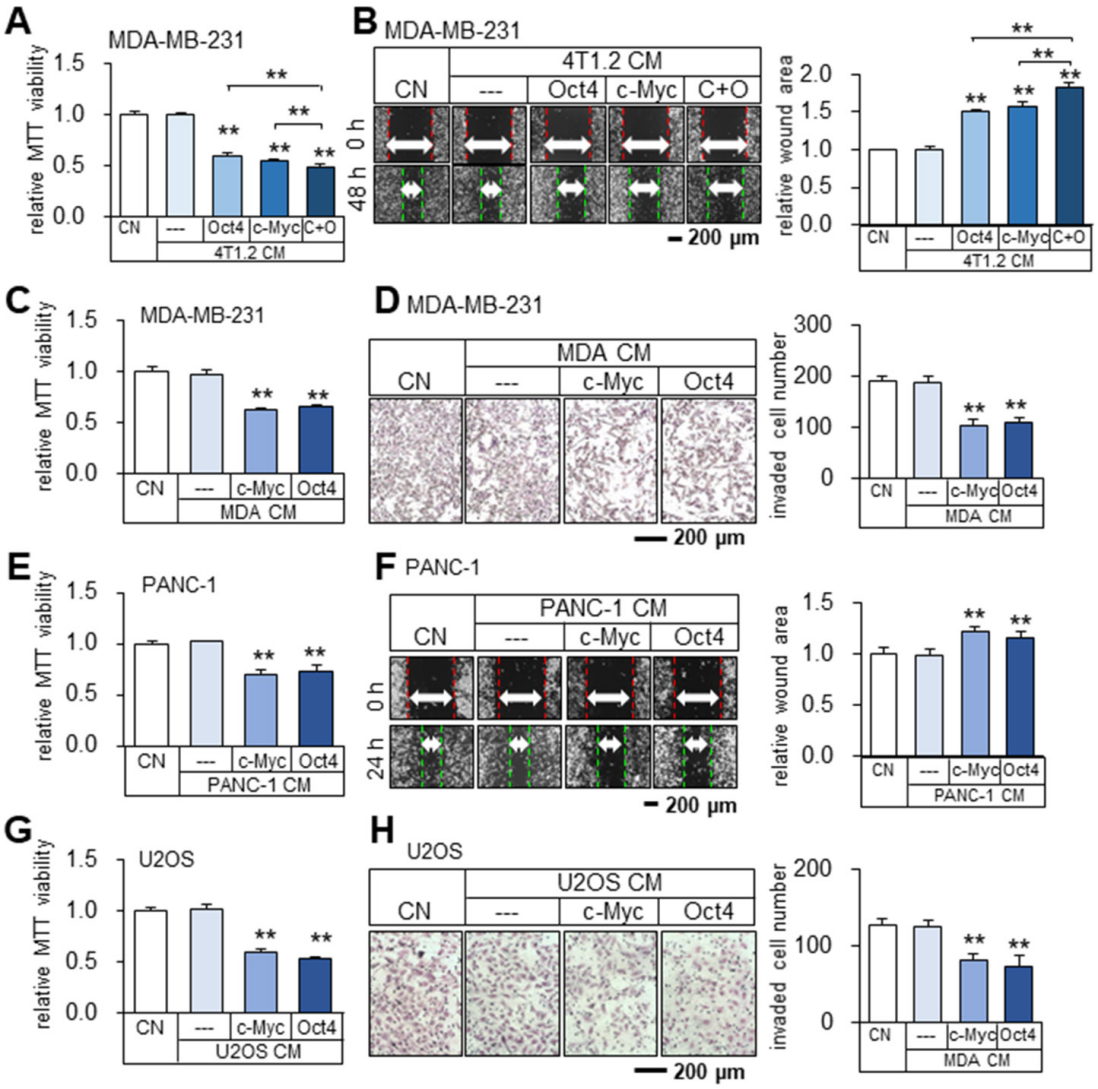
** Tumor-suppressing capability of Oct4- and c-Myc-overexpressing 4T1.2 tumor cell-derived CMs in MDA-MB-231 breast cancer cells, and inhibition of proliferation, migration, and invasion by tumor cell-derived iTS CM. CN = control, CM = conditioned medium, c-Myc = c-Myc plasmids, and Oct4 = Oct4 plasmids, and C+O = c-Myc plasmid + Oct4 plasmid.** The double asterisk indicates p < 0.01. (**A-B**) Reduction in MTT-based viability and scratch-based migration of MDA-MB-231 cells by Oct4- and c-Myc-overexpressing 4T1.2 tumor cell-derived CMs. (**C-D**) Inhibition of MTT-based viability and transwell invasion of MDA-MB-231 cells by c-Myc and Oct4-overexpressing MDA-MB-231 tumor cell-derived CM. (**E-F**) Inhibition of MTT-based viability and scratch-based migration of PANC-1 pancreatic cancer cells by c-Myc and Oct4-overexpressing PANC-1 tumor cell-derived CM. (**G-H**) Inhibition of MTT-based viability and transwell invasion of U2OS osteosarcoma cells by c-Myc and Oct4-overexpressing osteosarcoma cell-derived CM.

Besides 4T1.2 mammary tumor cells, the anti-tumor Oct4 CM, c-Myc CM, and Oct4/c-Myc CM were also derived from MDA-MB-231 breast cancer cells as well as EO771 mammary tumor cells (Figure 4A-B, Figure S4A-B). To further examine the possibility of generating iTS cells from other types of cancer cells, c-Myc or Oct4 was overexpressed in human and mouse cancer cell lines in the breast, pancreas, prostate, and bone. The result revealed that MDA-MB-231 breast cancer cell-derived CM inhibited MTT-based viability and transwell invasion of MDA-MB-231 breast cancer cells (Figure 4C-D). The same inhibitory responses were observed with the CMs derived from PANC-1 pancreatic cancer cells, U2OS osteosarcoma cells (Figure 4E-H), EO771 mammary tumor cells, and TRAMP-C2ras prostate tumor cells (Figure S4C-F).

### Undetectable tumor-suppressing effects of Sox2 and Klf4

**Figure 5:**
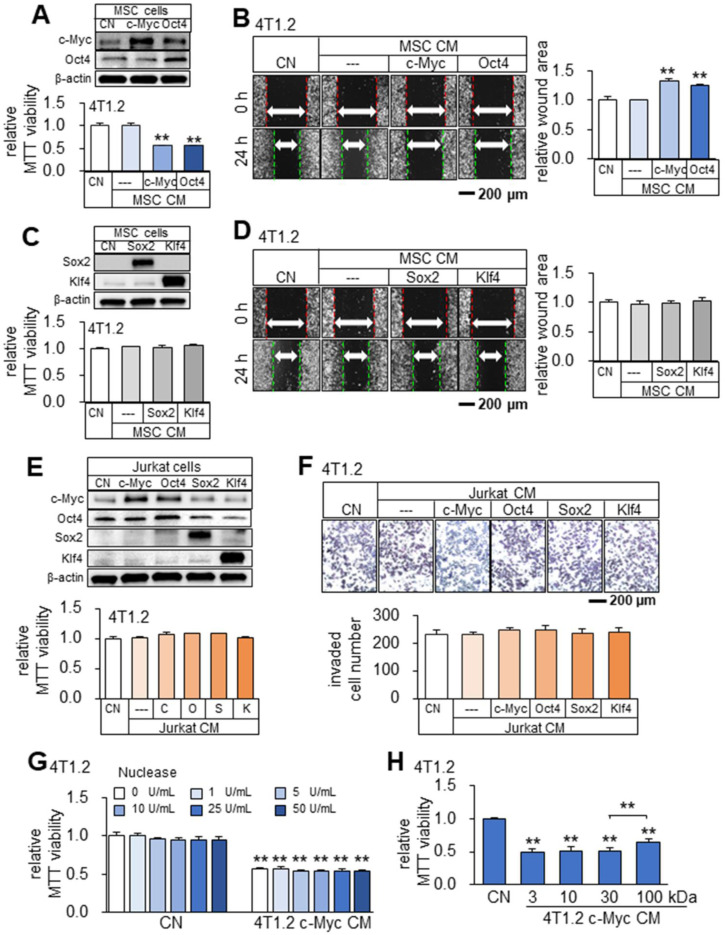
** Tumor-suppressing capability of Oct4- and c-Myc-overexpressing MSCs-derived CM in 4T1.2 mammary tumor cells.** The double asterisk indicates p < 0.01. CN = control, Oct4 = Oct4 plasmids, c-Myc = c-Myc plasmids, Sox2 = Sox2 plasmids, Klf4 = Klf4 plasmids, and CM = conditioned medium. (**A-B**) Elevation of c-Myc and Oct4 in MSC cells, and reduction in MTT-based viability and scratch-based migration of 4T1.2 cells by Oct4- and c-Myc-overexpressing MSC-derived CM. (**C-D**) Overexpression of Sox2 and Klf4, and no detectable effects on MTT-based viability and scratch-based migration in 4T1.2 cells by Sox2- or Klf4-overexpressing MSCs-derived CM. (**E-F**) No detectable effects on MTT-based viability and transwell invasion in 4T1.2 cells by c-Myc-, Oct4-, Sox2- and Klf4-overexpressing Jurkat cells-derived CM. (**G**) No significant nuclease-treatment effect to c-Myc-overexpressing 4T1.2-derived CM on MTT-based viability of 4T1.2 tumor cells. (**H**) Alterations in MTT-based viability of 4T1.2 cells by the protein fractions of c-Myc-overexpressing 4T1.2 tumor cell-derived CM. We employed the filters with the cut-off molecular weights of 3, 10, 30, and 100 kDa.

Besides tumor cells, we observed that CMs from Oct4- and c-Myc-overexpressing non-tumor cells (MSCs and MLO-A5 osteocytes) also presented anti-tumor capabilities (Figure 5A-B, Figure S5). While Oct4 and c-Myc made the secretomes of tumor cells and non-tumor cells tumor-suppressive, the overexpression of Sox2 or Klf4 did not show the anti-tumor capability. Sox2 and Klf4-overexpressing 4T1.2-derived CM did not induce detectable changes in MTT-based viability and scratch-based migration in 4T1.2, EO771, and MDA-MB-231 cell lines (data not shown). Besides, Sox2 and Klf4-overexpressing MSCs-derived CM also did not induce detectable changes in MTT-based viability and scratch-based migration in 4T1.2 mammary tumor cells (Figure 5C-D). Of note, Jurkat cell-derived CM, which was generated from the overexpression of c-Myc/Oct4 as well as Sox2/Klf4 did not induce detectable changes in MTT-based viability and transwell invasion in 4T1.2 mammary tumor cells (Figure 5E-F). Of note, since the nuclease treatment of c-Myc CM did not alter its anti-tumor effect (Figure 5G), we did not evaluate the potential effects of nucleic acids in the secretome. In the protein fractions of c-Myc CM, we observed that tumor-suppressing factors were widely distributed below and above 100 kDa (Figure 5H).

**Figure 6:**
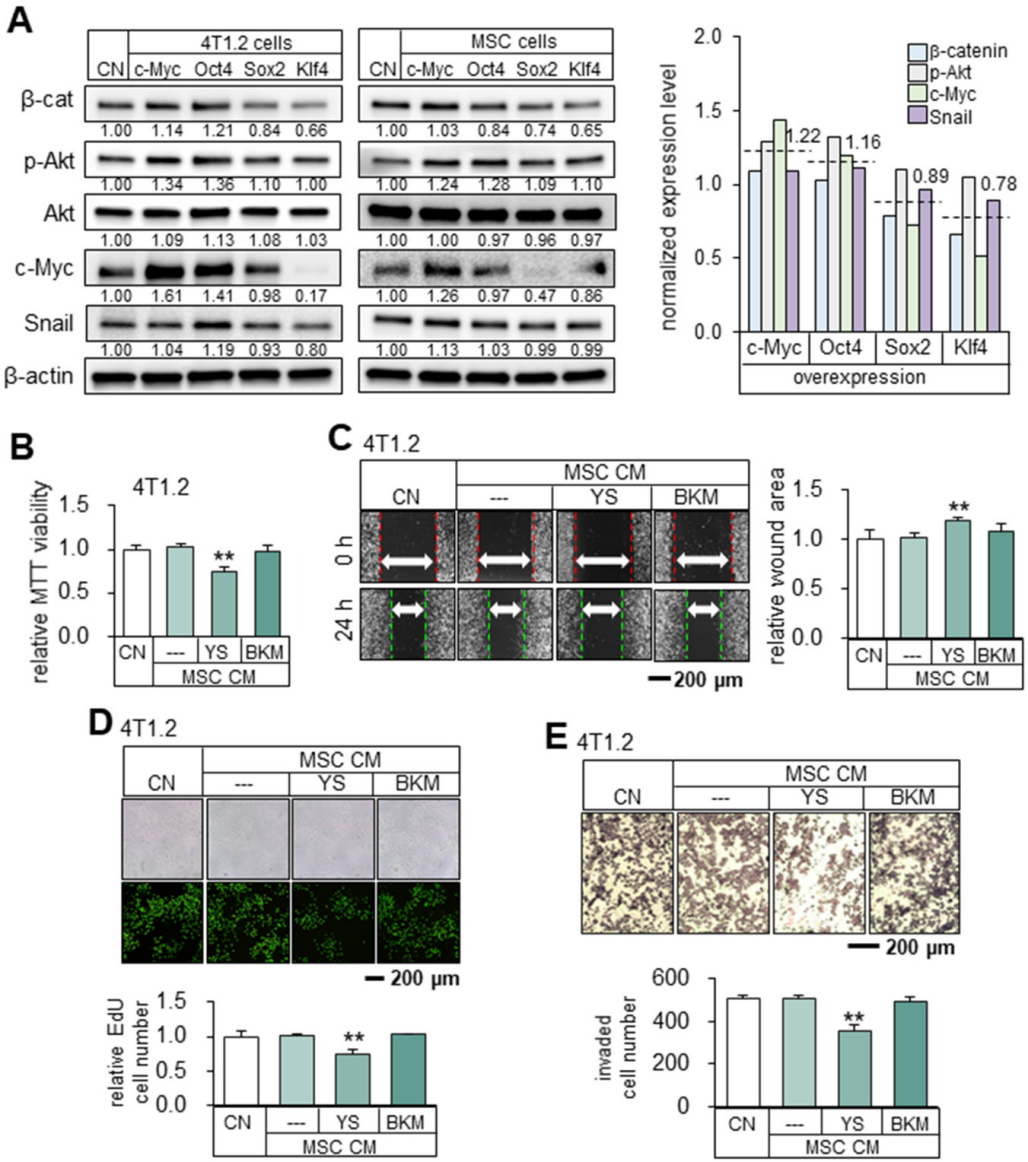
** Upregulation of β-catenin, p-Akt, c-Myc and Snail by the overexpression of c-Myc and Oct4 in 4T1.2 tumor cells.** The double asterisks indicate p < 0.01. CN = control, Oct4 = Oct4 plasmids, c-Myc = c-Myc plasmids, Sox2 = Sox2 plasmids, Klf4 = Klf4 plasmids, YS = YS49 (PI3K/Akt activator), BKM = BKM120 (PI3K inhibitor), and CM = conditioned medium. (**A**) Levels of β-cat, p-Akt, c-Myc and Snail in 4T1.2 cells and MSCs that overexpressed c-Myc, Oct4, Klf4, Sox2 or Klf4. (**B-C**) Reduction in MTT-based viability and scratch-based migration of 4T1.2 cells by YS49 (50 µM) treated MSC-derived CM, and no detectable effects by BKM (50 µM) treated MSC-derived CM. (**D-E**) Decrease in EdU-based proliferation and transwell invasion by YS49 treated MSC-derived CM, and no detectable effects by BKM treated MSC-derived CM.

Mechanistically, the overexpression of Oct4 and c-Myc elevated the expression of β-catenin, p-Akt, c-Myc, and Snail. In general, the average expression levels of β-cat, p-Akt, cMyc and Snail in 4T1.2 cells and MSCs are different between the two groups (c-Myc and Oct4) and (Sox2 and Klf4) (Figure 6A). The normalized mean levels of these 4 genes in 4T1.2 cells and MSCs were higher in the c-Myc/Oct4 group (1.22 and 1.16) than the Sox2/Klf4 group (0.89 and 0.78). To further explore the role of Akt, we employed YS49, a pharmacological agent for activating PI3K/Akt signaling. YS49-treated MSC-derived CM reduced the MTT-based viability, scratch-based migration, EdU-based proliferation, and transwell invasion of 4T1.2 cells, while no detectable effects were observed with a PI3K inhibitor, BKM-treated MSC-derived CM. (Figure 6B-E).

The overexpression of Oct4 and c-Myc in tumor cells reduced the expression of downstream oncogenic genes in parental tumor cells but the overexpression of Sox2 and Klf4 did not. We selected 6 protumorigenic genes (Kdm3a, Lrp5, MMP9, Runx2, TGFβ, and Snail) and evaluated their expression levels in response to CMs. Of note, Kdm3a is a histone demethylase to regulate the availability of chromatin, while Lrp5 is a co-receptor of Wnt signaling and MMP9 is a matrix metalloproteinase to promote tumor migration. Runx2 and TGFβ assist tumor progression, and Snail is involved in EMT. The result revealed that Oct4 CM and c-Myc CM downregulated all of these genes in 4T1.2 parental cells after 24-h incubation. Oct4/c-Myc CM (double transfection) further downregulated them, while Oct4 siRNA-treated CM suppressed the downregulation (Figure S6A-C). Also, OAC2 CM (a pharmacological Oct4 activator) presented the same inhibitory action (Figure S6D). Most importantly, however, no detectable change was observed in the expression of the selected genes by Sox2 CM and Klf4 CM (Figure S6E).

It should be noted that the overexpression of Oct4 induced the opposite effects in tumor cells vs. Oct4 CM-treated tumor cells. The selected protumorigenic genes (Lrp5, MMP9, Runx2, TGFβ, and Snail) were downregulated in Oct4 CM-treated 4T1.2 parental cells, while they were elevated in Oct4-overexpressing tumor cells and reduced in Oct4-silenced tumor cells (Figure S7). Collectively, Oct4 was oncogenic in Oct4-overexpressing tumor cells while it generated tumor-suppressive proteomes with Oct4-overexpressing tumor cells.

### Eno1, Hsp90ab1, Eef2, and vinculin as tumor suppressor candidates in CM

**Figure 7:**
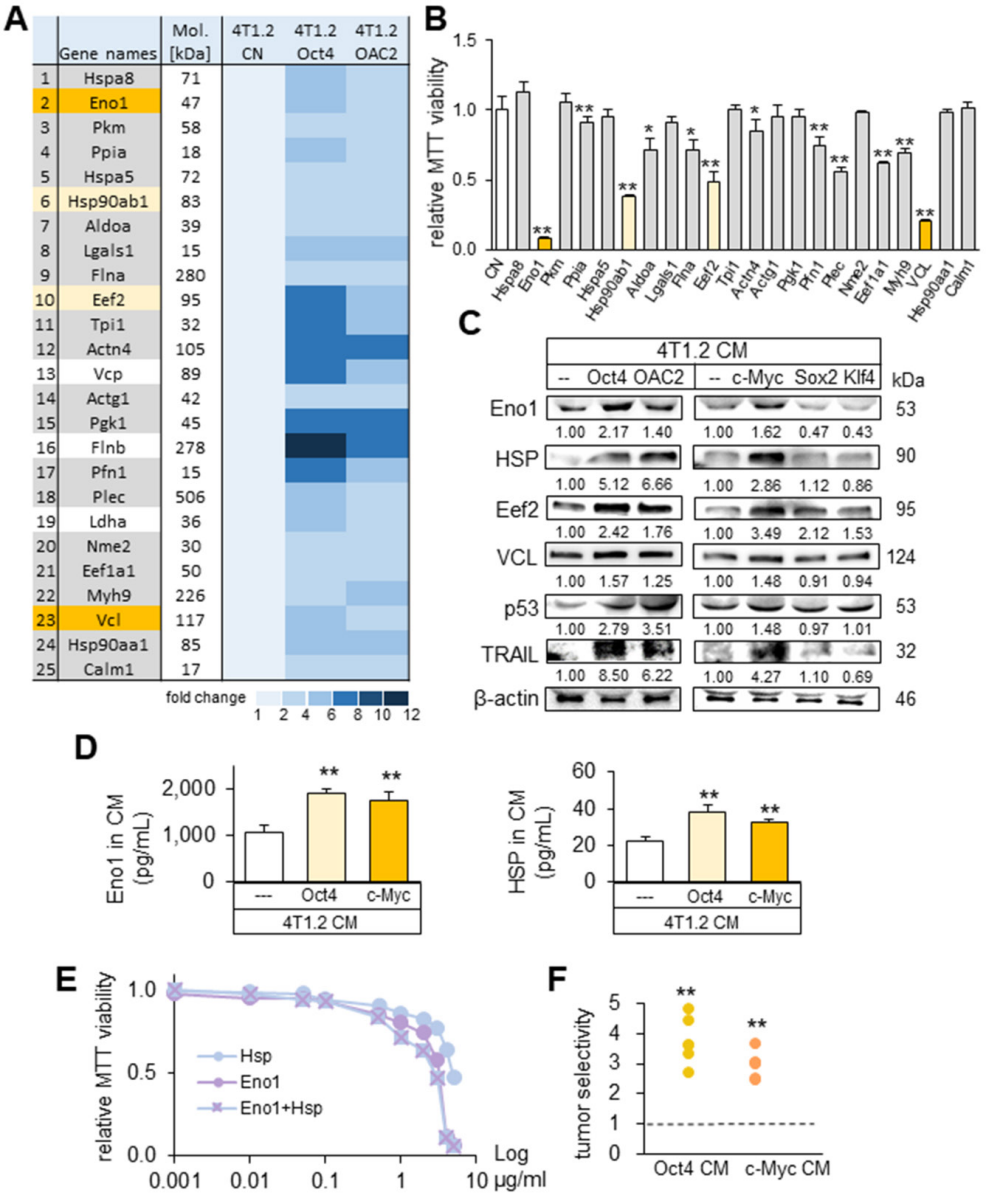
** Prediction of the tumor suppressors in CM by mass spectrometry-based whole-genome proteomics.** The single and double asterisks indicate p < 0.05 and 0.01, respectively. CN = control, Oct4 = Oct4 plasmids, c-Myc = c-Myc plasmids, Sox2 = Sox2 plasmids, Klf4 = Klf4 plasmids, and CM = conditioned medium. (**A**) Summary list of the potential tumor suppressors by mass spectrometry-based whole-genome proteomics. (**B**) Enolase 1 (Eno1), Hsp90ab1 (HSP), Eef2, and vinculin (VCL) as 4 tumor-suppressor candidates based on MTT-based viability. (**C**) Upregulation of Eno1, Hsp90ab1, Eef2, VCL, p53, and Trail in 4T1.2 cell-derived CM with the overexpression of Oct4, c-Myc, and the treatment with OAC2. The overexpression of Sox2 and Klf4 did not alter their levels. (**D**) Alterations in the levels of Eno1 and Hsp90ab1 in Oct4 and c-Myc CM by ELISA. (**E**) Reduction in MTT-based viability of 4T1.2 cells by the treatment with Eno1 and/or Hsp90ab1 recombinant proteins. (**F**) Tumor selectivity from the MTT-based viability of tumor cells (4T1.2 mammary tumor cells, EO771 mammary tumor cells, and MDA-MB-231 breast cancer cells) and human epithelium cells (KTB34-hTERT and KTB22-hTERT). Tumor selectivity is defined as a ratio of (MTT-based reduction in tumor cells) to (MTT-based reduction in non-tumor cells).

To determine the critical proteins for the tumor-suppressing action of Oct4 CM and OAC2 CM, mass spectrometry-based proteomics analysis was conducted. In the four CMs (medium control, CM control, Oct4 CM, and OAC2 CM), 395 proteins were identified in one of those CMs and 100 proteins were enriched in Oct4 CM and OAC2 CM (Table S1). As potential tumor suppressors, 25 candidates were selected and the effects of 22 proteins on the MTT-based viability of tumor cells were evaluated (Figure 7A). We observed that 12 proteins (5 µg/mL each) induced a statistically significant decrease in viability of 4T1.2 cells in 2 days (Figure 7B). The most striking reduction (50% or more) was detected with four proteins (Eno1, Hsp90ab1, Eef2, and vinculin). The expression levels of these putative tumor suppressors, together with p53 and Trail, were elevated in Oct4 CM, OAC2 CM, and c-Myc CM, but not in Sox2 CM and Klf4 CM (Figure 7C). Using ELISA, we detected the increased levels of Eno1 and Hsp90ab1 in Oct4 CM and cMyc CM (Figure 7D). The individual and combined efficacies of Hsp90ab1 and Eno1 recombinant proteins were plotted in the concentration range of 1 ng/mL to 5 µg/mL, showing their additive anti-tumor effects on MTT-based tumor viability (Figure 7E). The administration of CM, harvested from 4T1.2 cells that were treated with siRNAs specific to Eno1, Hsp90ab1, Eef2, and vinculin, elevated the transwell invasion of 4T1.2 cells (Figure S8A-B). Also, the scratch-based migration of 4T1.2 cells was stimulated and the levels of Lrp5, MMP9, Runx2, TGFβ, and Snail were elevated in 24 h (Figure S8C-D).

It is ideal if tumor-suppressing proteins selectively inhibit the progression of tumor cells without affecting the growth of non-tumor cells. Using the 2-day MTT-based viability assay, we determined tumor selectivity, a parameter defined as a ratio of (viability reduction of tumor cells) to (viability reduction of non-tumor cells). The tumor selectivity should be above one to preferentially remove tumor cells and the larger value is desirable to reduce the metabolic inhibition of non-tumor cells. In this assay, we employed three tumor cell lines (4T1.2 mouse mammary tumor cells, EO771 mammary tumor cells, MDA-MB-231 breast cancer cells) and two human control epithelium cells (KTB34-hTERT and KTB22-hTERT). The result showed that the inhibitory effects of both Oct4 CM and c-Myc CM were selective to tumor cells, and Oct4 CM presented a higher selectivity than c-Myc CM (Figure 7F).

### Differential roles of intracellular and extracellular Eno1, Hsp90ab1, Eef2, and vinculin

**Figure 8:**
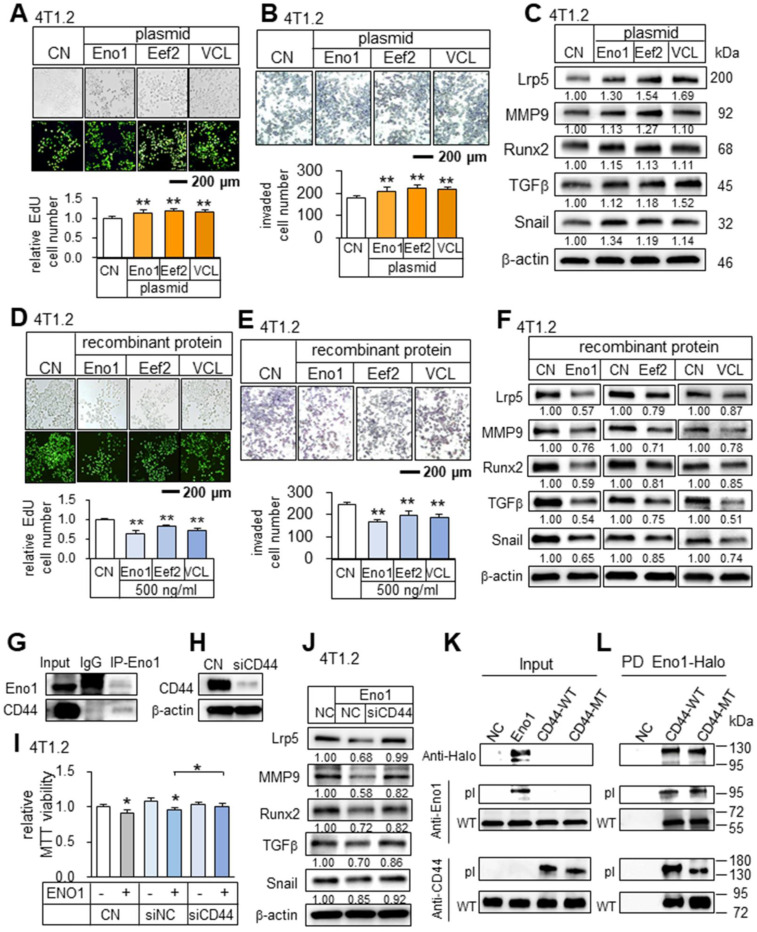
** Tumor-promoting effects by the overexpression of Eno1, Eef2, and VCL in 4T1.2 mammary tumor cells, and tumor-suppressing effects by the administration of their recombinant proteins.** The double asterisk indicates p < 0.01. CN = control, Eno1 = enolase 1, VCL = vinculin, and Hsp = Hsp90ab1. (**A-C**) Elevation in EdU-based proliferation, transwell invasion, and the upregulation of Lrp5, MMP9, Runx2, TGFβ, and Snail by the overexpression of Eno1, Eef2, and VCL in 4T1.2 tumor cells. (**D**) Decrease in EdU-based proliferation by the administration of Eno1, Eef2, and VCL recombinant proteins. (**E**) Reduction in transwell invasion by the administration of Eno1, Eef2, and VCL recombinant proteins. (**F**) Downregulation of Lrp5, MMP9, Runx2, TGFβ, and Snail in 4T1.2 tumor cells by the administration of Eno1, Eef2, and VCL recombinant proteins. (**G**) Co-immunoprecipitation of CD44 by Eno1 in 4T1.2 cells. (**H-I**) Suppression of the reduction in MTT-based viability by Eno1 in response to the silencing of CD44. (**J**) Downregulation of Lrp5, Runx2, MMP9 and Snail in 4T1.2 cells by the administration of Eno1 recombinant proteins, and the suppression by the silencing of CD44. (**K-L**) Western blotting of wild-type and mutant CD44 proteins, which were pulled down by Halo-tagged Eno1 proteins. PD = pull-down assay, NC = negative control, pl = Eno1 or CD44 proteins from plasmid transfection, MT = mutant CD44 without a cytoplasmic domain, and WT = wild-type CD44.

In analyzing the role of the predicted tumor suppressors, we examined their intracellular and extracellular roles. The overexpression of Eno1, Eef2, and vinculin in 4T1.2 cells elevated the EdU-based proliferation and transwell invasion of 4T1.2 cells, as well as the levels of Lrp5, MMP9, Runx2, TGFβ, and Snail (Figure 8A-C). However, the application of their recombinant proteins (0.5 μg/mL) extracellularly to 4T1.2 cells reduced the EdU-based proliferation, transwell invasion, and downregulated Lrp5, MMP9, Runx2, TGFβ, and Snail (Figure 8D-F). Interestingly, while p53 is known to serve as a tumor suppressor intracellularly, we observed that CMs derived from p53-overexpressing 4T1.2 cells and MLO-A5 osteocytes also presented the anti-tumor capabilities (Figure S9A-B). Regarding Hsp90ab1, the application of its recombinant proteins reduced transwell invasion and downregulated Lrp5, MMP9, Runx2, TGFβ, and snail (Figure S9C-D). Taken together, the result showed that the identified tumor-suppressing proteins were moonlighting proteins with differential roles in the intracellular and extracellular domains.

### Potential binding partners of Eno1

**Figure 9:**
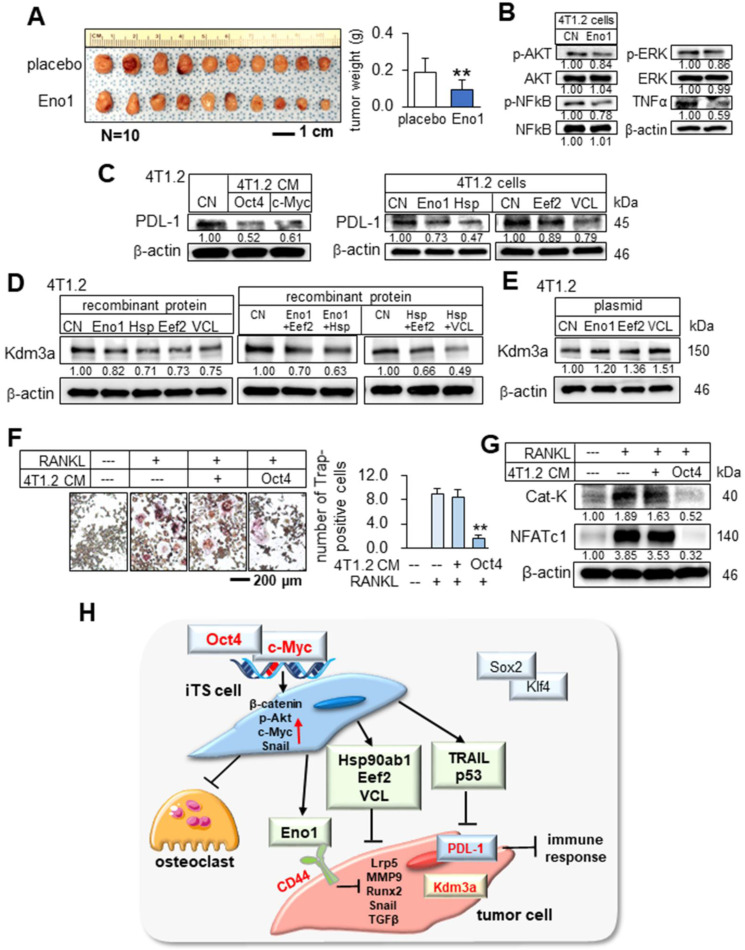
** Downregulation of PDL-1 and Kdm3a in 4T1.2 mammary tumor cells by Hsp90ab1, Eno1, Eef2, and VCL, and the suppression of the development of osteoclasts.** CN = control, Hsp = Hsp90ab1, Eno1 = enolase 1, VCL = vinculin, Oct4 = Oct4 plasmids, and CM = conditioned medium. The double asterisk indicates p < 0.01. (**A**) Reduction in the size and weight of mammary tumors by the daily intravenous administration of 1 μg/mL Eno1. EO771 mammary tumor cells were inoculated into the mammary fat pad of C57BL/6 female mice (N = 10). **(B)** Reduction in p-AKT, NFkB p65, p-ERK, and TNFα in 4T1.2 cells in response to Eno1 recombinant proteins. (**C**) Reduction in PDL1 in 4T1.2 cells by Oct4/cMyc-overexpressing 4T1.2 CM, and Eno1, Hsp, Eef2, and VCL recombinant proteins. (**D**) Reduction in Kdm3a in 4T1.2 cells in response to Eno1, Hsp90ab1, Eef2, and/or VCL recombinant proteins. (**E**) Elevation of Kdm3a in 4T1.2 cells by the overexpression of Eno1, Eef2, and VCL. (**F**) Suppression of RANKL-stimulated osteoclast development by Oct4-overexpressing CM. (**G**) Reduction in the levels of cathepsin K and NFATc1 in RANKL-stimulated osteoclasts by Oct4-overexpressing CM. (**H**) Regulatory mechanism by the tumor-suppressive secretomes, which were derived from Oct4- and c-Myc-overexpressing tumor cells.

To assess the potential mechanism of the tumor-suppressive action of Eno1, we conducted an immunoprecipitation assay. The result revealed that in the protein extracts from 4T1.2 cells CD44 was co-immunoprecipitated with Eno1 (Figure 8G). Notably, Eno1-driven reduction in MTT-based viability and the levels of tumorigenic genes (Lrp5, MMP9, Runx2, TGFβ, and Snail) was suppressed when CD44 was silenced (Figure 8H-J). To further examine the interactions of Eno1 with CD44, we conducted a pull-down experiment using a Halo-tagged Eno1 protein. Particularly, to evaluate the role of cytoplasmic and extracellular domains of CD44, we employed wild-type CD44 (WT CD44) and mutant CD44 (MT CD44) that lacked a cytoplasmic domain. The results using 4T1.2 mammary tumor cells showed that Eno1 pulled down both WT and MT CD44 proteins, indicating that extracellular Eno1 interacted with the ECM domain of CD44 (Figure 8K-L). Furthermore, the daily administration of Eno1 proteins at 1 µg/kg significantly reduced the growth of mammary tumors of C57BL/6 female mice in 2 weeks (Figure 9A). Notably, the incubation of 4T1.2 cells with 0.5 µg/mL Eno1 recombinant proteins reduced the levels of the phosphorylated form of Akt, NFκB p65, and Erk, as well as TNFα (Figure 9B).

### Suppression of PDL-1 by c-Myc and Oct4 CMs

We examined the effect of c-Myc and Oct4 CMs on T cell-linked immune responses that were mediated by the interactions of PDL-1 with PD-1. Of note, PDL-1 in tumor cells is a ligand of PD-1 and it inactivates immune responses by T cells. Notably, the expression of PDL-1 was downregulated in 4T1.2 mammary tumor cells by c-Myc and Oct4-overexpressing 4T1.2 CM in 24 h, as well as the incubation with Eno1, Hsp90ab1, Eef2, and vinculin (Figure 9C). The result indicates that c-Myc and Oct4 CMs and the newly identified tumor-suppressing proteins can act as PDL-1 inhibitors.

### Kdm3a as a target of Eno1, Hsp90ab1, Eef2, and vinculin

We have shown that the expression of Kdm3a, lysine-specific demethylase for histones, in 4T1.2 tumor cells was downregulated by Oct4 CM and c-Myc CM. Consistently, the administration of Eno1, Hsp90ab1, Eef2, and vinculin at 0.5 µg/mL also downregulated Kdm3a in 4T1.2 cells. For a limited set of cases, we examined the effect of a combinatorial administration of these proteins and observed the enhanced reduction in Kdm3a (Figure 9D). By contrast, the overexpression of Eno1, Eef2, and vinculin in 4T1.2 cells elevated the level of Kdm3a (Figure 9E). The result suggests that histone methylation is altered by the newly identified extracellular tumor suppressors, which were enriched in CM by the overexpression of Oct4 and c-Myc and not by the overexpression of Sox2 and Klf4.

### Suppression of osteoclast development

In tumor-invaded bone, bone-resorbing osteoclasts play a major role in bone destruction, and blocking their development is critically important. We examined the effect of Oct4 CM on the maturation of RAW264.7 pre-osteoclasts. The result revealed that Oct4 CM inhibited the differentiation of RANKL-stimulated pre-osteoclasts and downregulated two key regulators, cathepsin K as a bone-resorbing protease and NFATc1 as a master transcription factor of osteoclastogenesis in 24 h (Figure 9F-G). The result indicates that the described secretome may repress the vicious bone-degradation cycle in the bone microenvironment by reducing not only tumor progression but also osteoclast development.

## Discussion

We presented in this study that two of the Yamanaka factors, Oct4 and c-Myc, can be used to convert the secretome of tumor cells into tumor-suppressive, while two other factors, Sox2 and Klf4, failed to induce tumor-suppressing capabilities. The application of Oct4 CM, c-Myc CM, and Oct4/c-Myc CM to tumor cells reduced the proliferation, migration, invasion, and growth of three-dimensional tumor spheroids, and in the mouse models, the systemic administration of CMs inhibited the growth of mammary tumors and bone degradation. These newly identified tumor suppressors, such as extracellular Eno1, Hsp90ab1, Eef2, and vinculin, downregulated Kdm3a, a histone demethylase, as well as the downstream oncogenic genes 21-24. In contrast, Sox2 CM and Klf4 CM did not alter the expression of Kdm3a and the selected oncogenic genes. Besides, c-Myc and Oct4 CMs and the newly identified tumor-suppressing proteins downregulated PDL-1, a target of anti-PD1 immunotherapy 25. Collectively, the results indicated that the overexpression of Oct4 and c-Myc (and not Sox2 or Klf4) in tumor cells elevates the extracellular levels of Eno1, Hsp90ab1, Eef2, and vinculin and the application of Oct4 CM, c-Myc CM, and Oct4/c-Myc CM to tumor cells and tumor-bearing mice inhibited the growth of tumors (Figure 9H).

Importantly, iTS cells can be generated not only from breast cancer cells, but also from prostate cancer, pancreatic cancer, and osteosarcoma cells, as well as MSCs and osteocytes. In our previous studies, iTS cells were also generated from MSCs by overexpressing Lrp5, β-catenin, Snail, or Akt 3, from osteocytes by overexpressing Lrp5 and β-catenin 5, 6, and from tumor cells by overexpressing β-catenin 26. Notably, all iTS cells, which have been generated so far, inhibit not only the progression of tumors but also the development of osteoclasts and prevent the bone-destroying vicious cycle in the tumor-bone microenvironment. While T-lymphocytes were unable to be converted into iTSCs by overexpressing c-Myc and Oct4 in this study, we previously showed that they became iTSCs by activating PKA signaling 27. Taken together, the overexpression of c-Myc and Oct4 does not guarantee the production of tumor-suppressive proteomes and the genes to be activated may depend on types of iTSC-generating cells.

Existing data suggest that the removal of tumor cells by iTSCs might result from local and remote cell competition 1. In Drosophila organogenesis and mouse embryos, cells with higher protein synthesis are considered capable of eliminating neighboring cells 16, 28. Although the mechanism of actions of iTSCs and its linkage to cell stemness is still to be elucidated, the upregulation of cell-proliferating signalings such as Wnt and PI3K was associated with the overexpression of c-Myc and Oct4 in this study. While all Yamanaka factors are necessary to generate iPS cells, iTS cells were produced by the overexpression of Oct4 or c-Myc alone 29. We observed that histone demethylase, Kdm3a, was downregulated in tumor cells treated with Oct4 CM and c-Myc CM, but it was not altered by the treatment with Sox2 CM and Klf4 CM. Kdm3a is a key regulator of cancer stemness by modifying chromatin structure and transcriptional efficiency 30.

Individual proteins can have multiple functions based on their context. Oct4 and c-Myc in this study are examples of such moonlighting proteins with opposing tasks inside and outside of their overexpressing cells. It is reported that Oct4 not only maintains pluripotency in embryonic cells but also regulates the proliferation of cancer cells 31, 32. The upregulation of c-Myc has been observed in many cancers 10. In MCF-7 breast cancer cells, c-Myc is reported to inhibit tumor migration, while in MDA-MB-231 cells it is reported to promote migration 33. This study shows that the intracellular environment of Oct4-overexpressing tumor cells is tumor-promoting, while their extracellular environment is tumor-suppressing. It should be noted that tumor cell-derived exosomes may contain tumor-promoting factors 34 and we removed exosomes from CMs by the ultracentrifugation. However, CMs were tumor-suppressive without the ultracentrifugation.

It was unexpected that the proteins such as Hsp90ab1, Eno1, Eef2, and vinculin were enriched in the secretome since they are considered tumor promoters intracellularly 35-38. Most notably, our analyses using the overexpression and recombinant proteins revealed that these proteins extracellularly act as tumor suppressors while intracellularly as tumor promoters. Silencing these proteins reduced the extracellular level of p53 39, 40 and Trail 41, 42, which are known as a tumor suppressor and an apoptosis inducer selective to tumor cells, respectively. Regarding the action of extracellular Hsp90ab1, it may impede the activation of TGFβ that is inhibited by latency-associated protein (LAP). Hsp90ab1 is reported to block the removal of LAP from the TGFβ-LAP complex 43.

Regarding Eno1, its truncated form, Myc promoter-binding protein 1 (MBP1), is known to repress the transcription of c-Myc 44. While we showed that extracellular Eno1 downregulated c-Myc (Figure S9E) as well as p-Akt, p-p65, and p-Erk in tumor cells, it is unclear whether it may act as MBP1. The immunoprecipitation result indicated that extracellular Eno1 interacted with a membrane-bound CD44 in tumor cells and suppressed the progression of tumor cells. CD44 is a transmembrane protein, whose N-terminal extracellular domain contains a motif serving as a docking site 45. The pull-down result using mutant CD44 proteins in this study was consistent with the role of the extracellular domain of CD44 in the interaction with extracellular Eno1. CD44 supports signaling that not only inhibits but also promotes cancer progression 19. It is necessary to evaluate whether the Eno1-CD44 axis is always inhibitory or context-dependent. Vinculin has been shown to interact with E-cadherin, which can act as a tumor suppressor 19, 46, 47. It may also interact with Sorbin that is reported to suppress metastasis 48, 49. While a kinase for Eef2 was reported to act as a tumor suppressor as well as a stimulator 50, 51, to the best of our knowledge, no reports were available for possible anti-tumor action of Eef2. Since atypical tumor-suppressing proteins presented a dichotomous role, inhibiting their whole activity, for instance in chemotherapy, may reduce their tumor-suppressing capability in the extracellular domain.

The efficacy and selectivity may depend on the types of initiating tumor cells and targeted tumor cells. It is important to determine an effective size range of anti-tumor protein fractions, and the results indicated that tumor suppressors were widely distributed below and above 100 kDa. We also evaluated the dose responses of Eno1 and/or Hsp90ab1 using their recombinant proteins and observed their additive effects in a wide concentration range. Further analyses are necessary to evaluate the frequency and administrative routes of a single or combinatorial application of these tumor-suppressing proteins. Although the role of other entities such as RNA and DNA in the anti-tumor and bone-protective secretors cannot be ignored, this study mainly explored protein-based secretomes since the use of nucleases did not significantly alter the anti-tumor capability of CM. The result herein also casts light on the inconsistent outcomes of surgical removal of primary cancers, which have puzzled clinicians and oncologists for over a century 20. While physical elimination is in general considered a vital intervention for patients with cancer, it may entail adverse outcomes such as the acceleration of the growth of residual tumors and metastases. This clinical observation is known as concomitant tumor resistance, in which a tumor-bearing host is resistant to the growth of secondary tumors 52. As a cause of concomitant tumor resistance, trauma, local and systemic inflammatory, and the enhanced nutrient supply to remaining tumors are contemplated. Besides the above factors, this study supports the notion that the discontinued supply of tumor-suppressive secretory factors from the excised tumor may contribute to the accelerated growth and migration of residual tumors 53.

In summary, this study demonstrates that the secretome of tumor cells can be converted into tumor-suppressive agents by overexpressing the transcription factors such as Oct4 and c-Myc. The administration of CM strikingly inhibited the growth of mammary tumors and tumor-induced osteolysis in the mouse model. Of note, immunocompatible mice were employed in this study, but the previous study from our group has shown that MSC-derived iTSC CM has anti-tumor capability in the immunocompromised mouse model 4. This study provides multiple pieces of evidence to support the anti-tumor actions of tumor cell-derived secretomes. Unlike their cohort tumor cells without any overexpression, we observed that Oct4 and c-Myc-overexpressing tumor cells were resistant to their CM. Ironically, the activation of tumor progression generated tumor cell-derived tumor-suppressive secretomes. We expect that the described tumor-driven secretomes and the newly identified tumor-suppressing proteins may provide a novel therapeutic option for breast cancer and associated bone metastasis. Developing oncogenic activators might become a unique task for pharmaceutical industries to generate potent anti-tumor agents.

## Materials and methods

### Cell culture

EO771 mouse mammary tumor cells (CH3 BioSystems, Amherst, NY, USA), 4T1.2 mouse mammary tumor cells (obtained from Dr. R. Anderson at Peter MacCallum Cancer Institute, Melbourne, Australia), PANC-1 human pancreatic cancer cells (ATCC, Manassas, VA, USA), and U2OS osteosarcoma cells (Sigma, St. Louis, MO, USA) were cultured in DMEM. MDA-MB-231 breast cancer cells (ATCC), RAW264.7 pre-osteoclast cells (ATCC), and MLO-A5 osteocyte-like cells (C57BL/6 background; obtained from Dr. L. Bonewald at Indiana University, IN, USA) were grown in αMEM. Jurkat T lymphocytes were cultured in RPMI1640. TRAMP-C2ras prostate tumor cells (ATCC) were cultured in DMEM/F-12. Human epithelium cells (non-tumor immortalized control cells; KTB34-hTERT and KTB22-hTERT obtained from Dr. Nakshatri, Indiana University, IN, USA) were cultured in F12 and low glucose DMEM (3:1) with hydrocortisone (0.4 µg/mL, #H0888, Sigma), insulin (5 µg/mL, #I5500, Sigma) and EGF (20 ng/mL, #236-EG-200, R&D systems, Minneapolis, MN, USA). Murine mesenchymal stem cells (MSCs) derived from the bone marrow of the C57BL/6 strain (Envigo RMS, Inc., Indianapolis, IN, USA) were cultured in MesenCult culture medium (Stem Cell Technology, Cambridge, MA, USA). The culture media was supplemented with 10% fetal bovine serum and antibiotics (100 units/mL penicillin, and 100 µg/mL streptomycin; Life Technologies, Grand Island, NY, USA), and cells were maintained at 37 °C and 5% CO_2_.

### Preparation of CM

CM was subjected to low-speed centrifugation at 2,000 rpm for 10 min. The cell-free supernatants were centrifuged at 4,000 rpm for 10 min and subjected to filtration with a 0.22-μm polyethersulfone membrane (Sigma). The supernatants were further centrifuged at 10,000 × g for 30 min at 4 °C to remove remaining cell debris and at 100,000 × g (Type 90 Ti Rotor, Beckman, Brea, CA, USA) overnight at 4 °C to remove exosomes. To evaluate the effect of nucleic acids on the anti-tumor action of iTS-CM, we treated iTS-CM with nucleases for digesting DNA and RNA (PI88700, Thermo Fisher Scientific, Waltham, MA, USA). We also used four filters with different cutoff weights of 3, 10, 30, and 100 kDa (#UFC900324, #UFC8010, #UFC8030, #UFC8100, Sigma) and evaluated the anti-tumor efficacy of the size-fractionated CMs.

### EdU assay

Using the procedure previously reported 54, approximately 1,000 cells were seeded in 96-well plates on day 1. CM was added on day 2 and cellular proliferation was examined with a fluorescence-based cell proliferation kit (Click-iT™ EdU Alexa Fluor™ 488 Imaging Kit; Thermo Fisher Scientific) on day 4. After fluorescent labeling, the number of fluorescently labeled cells was counted and the ratio to the total number of cells was determined.

### Transwell invasion assay

In a transwell invasion assay, approximately 5×10^4^ cells in 200 µL serum-free DMEM were placed on the upper transwell chamber (Thermo Fisher Scientific) with Matrigel (100 µg/mL), and 800 µL of CM was added in the lower chamber. After 48 h, the cells that had invaded the lower side of the membrane were stained with Crystal Violet. At least five randomly chosen images were taken, and the average number of stained cells was determined.

### Scratch assay

A wound-healing scratch assay was conducted to evaluate 2-dimensional migratory behavior. Approximately 4×10^5^ cells were seeded in 12-well plates. After cell attachment, a plastic pipette tip was used to scratch a gap on the cell layer. The floating cells were removed and CM was added. Images of the cell-free scratch zone were taken at 0 h, and the areas newly occupied with cells were determined 24-48 h after the scratching. The areas were quantified with Image J (National Institutes of Health, Bethesda, MD, USA).

### Osteoclast differentiation assay

The differentiation assay of RAW264.7 pre-osteoclasts was performed in a 12-well plate. During the 6-day incubation of pre-osteoclast cells in 40 ng/mL of RANKL, the culture medium was exchanged once on day 4. Adherent cells were fixed and stained with a tartrate-resistant acid phosphate (TRAP)-staining kit (Sigma), according to the manufacturer's instructions. TRAP-positive multinucleated cells (> 3 nuclei) were identified as mature osteoclasts.

### Western blot analysis

Cultured cells were lysed in a radio-immunoprecipitation assay buffer and proteins were fractionated by 10-15% SDS gels and electro-transferred to polyvinylidene difluoride transfer membranes (Millipore, Billerica, MA, USA). The membrane was incubated overnight with primary antibodies and then with secondary antibodies conjugated with horseradish peroxidase (Cell Signaling, Danvers, MA, USA). Antibodies against c-Myc, Oct4, Sox2, Klf4, Lrp5, Runx2, Snail, TGFβ, Eno1, Eef2, vinculin, β-catenin, p-Akt, Akt, p-NFkB p65, NFkB p65, p-ERK, ERK (Cell Signaling), MMP9, NFATc1, cathepsin K (Santa Cruz, Dallas, TX, USA), TRAIL (Novus, Centennial, CO, USA), p53 (Invitrogen, Carlsbad, CA, USA), Hsp90ab1 (Abcam, Cambridge, UK), Kdm3a (Thermo Fisher Scientific) and β-actin as a control (Sigma) were employed. The level of proteins was determined using a SuperSignal west femto maximum sensitivity substrate (Thermo Fisher Scientific), and a luminescent image analyzer (LAS-3000, Fuji Film, Tokyo, Japan) was used to quantify signal intensities.

### Plasmid transfection, RNA interference, and ELISA

The overexpression of c-Myc, Oct4, Sox2, Klf4, Eno1, vinculin, and Eef2 was conducted by transfecting plasmids (#17758, #19778, #26817, #26815, #27563, #58198; Addgene, Cambridge, MA, USA; and HG13966-G, Sino Biological, Wayne, PA, USA), while blank plasmids (FLAG-HA-pcDNA3.1; Addgene) were used as a control. RNA interference was conducted using siRNA specific to Oct4, Eno1, Hsp90ab1, Eef2, vinculin, and CD44 (115304, s234544, s67897, 157269, 186995, s63659; Thermo-Fisher) with a negative siRNA (Silencer Select #1, Thermo Fisher Scientific) as a nonspecific control using the procedure previously described 55. The expression levels of Eno1 and Hsp90ab1 in CM were detected by ELISA (MyBioSource, San Diego, CA, USA).

### 3D spheroid competition assay and* ex vivo* tissue assay

In a three-dimensional spheroid assay, tumor spheroids were formed by culturing cells in the U-bottom low-adhesion 96-well plate (S-Bio, Hudson, NH, USA) at 1×10^4^ 4T1.2 cells/well. To evaluate the effect of secretome, the medium was replaced by CM. Fluorescently labeled 4T1.2 cells were prepared by culturing them with a green (#4705, Sartorius, Gottingen, Germany) or red fluorescent dye (#4706) for 20 min at 37 °C. Cells were then harvested as a pellet by centrifuging at 1,000 rpm for 5 min. Cells were imaged every 24 h, and the area was calculated with Image J.

In the *ex vivo* tissue assay, the usage of human breast cancer tissues was approved by the Indiana University Institutional Review Board, and the tissues were received from Simon Cancer Center Tissue Procurement Core. A sample (~ 1 g) was manually fragmented with a scalpel into small pieces (0.5 ~ 0.8 mm in length), which were grown in DMEM with 10% fetal bovine serum and antibiotics for a day. CM was then added for two additional days and a change in the fragment size was determined.

### Immunoprecipitation

Protein samples were pretreated with agarose beads conjugated with protein A and rabbit IgG, followed by the overnight immunoprecipitation with the beads conjugated with anti-Eno1 antibodies (Cell Signaling). The beads were collected by centrifugation, washed three times with PBS, and resuspended for Western blotting. Western blotting was conducted using antibodies against Eno1 and CD44 (Cell Signaling).

### Pull-down assay

The protein lysates were prepared from 4T1.2 cells, which were transfected with GFP-tagged wild-type CD44 plasmids (#137823, Addgene), GFP-tagged mutant CD44 plasmids lacking the cytoplasmic domain (#137822, Addgene), or Halo-tagged Eno1 plasmids (#175330, Addgene). The lysates were mixed with the beads conjugated with anti-Halo antibodies overnight (Promega, Madison, WI, USA). The beads were then collected by centrifugation and washed three times with PBS, followed by immunoblotting with anti-Halo, anti-Eno1, and anti-CD44 antibodies.

### Animal models

The experimental procedures were approved by the Indiana University Animal Care and Use Committee and were complied with the Guiding Principles in the Care and Use of Animals endorsed by the American Physiological Society. Mice were randomly housed five per cage by a stratified randomization procedure based on body weight. Mouse chow and water were provided *ad libitum*. We employed BALB/c female mice with 4T1.2 mammary tumor cells and C57BL/6 female mice with EO771 mammary tumor cells. BALB/c female mice (~8 weeks, Envigo) were divided into 3 groups (placebo, Oct4 CM, and OAC2 CM groups; 8 mice per group) in the first experiment, while they were divided into 3 groups (placebo, c-Myc CM, and Oct4/c-Myc CM groups; 12 mice per group) in the second experiment. In the mouse model of a mammary tumor, BALB/c mice received a subcutaneous injection of 4T1.2 cells (3.0 × 10^5^ cells in 50 µL PBS) to the mammary fat pad on day 1 56. For the tibial osteolysis mouse model, BALB/c mice received an intra-tibial injection of 4T1.2 cells (3.0 × 10^5^ cells in 20 µL PBS) to the right tibia on day 1. For examining the efficacy of Eno1, C57BL/6 female mice (~8 weeks, Envigo) were divided into 2 groups (placebo and Eno1 groups; 10 mice per group) and received a subcutaneous injection of EO771 mammary tumor cells (3.0 × 10^5^ cells in 50 µL PBS) to the mammary fat pad on day 1. From day 2, mice were given a daily intravenous injection of PBS (placebo group) or 1 µg/mL of Eno1 (Eno1 group). The animals were sacrificed on day 14, and the tumor weight was determined.

For examining the efficacy of Oct4- and c-Myc-overexpressing CMs *in vivo*, fetal bovine serum-free CM was condensed by a filter (#UFC9003, Amicon^®^ Ultra-15 Centrifugal Filter Unit, Sigma) with a cutoff molecular weight of 3 kDa and the 10-fold condensed CM (50 μL re-suspended in PBS) was intravenously injected from the tail vein at the same time from day 2. The animals were sacrificed on day 14 and mammary tumors and tibiae were harvested.

### μCT imaging and histology

The tibiae were harvested for μCT imaging and histology. Micro-computed tomography was performed using Skyscan 1172 (Bruker-MicroCT, Kontich, Belgium). Using manufacturer-provided software, scans were performed at pixel size 8.99 μm and the images were reconstructed (nRecon v1.6.9.18) and analyzed (CTan v1.13). Using µCT images, trabecular bone parameters such as bone volume ratio (BV/TV), bone mineral density (BMD), trabecular number (Tb.N), and trabecular separation (Tb.Sp) were determined in a blinded fashion. In histology, H&E staining was conducted as described previously and images were analyzed in a blinded fashion 57. Of note, normal bone cells appeared in a regular shape with round and deeply stained nuclei, while tumor cells were in a distorted shape with irregularly stained nuclei.

### Mass spectrometry-based proteomics analysis

The freeze-dried pellet samples were harvested from the culture medium using 4T1.2 mouse mammary tumor cells, which were treated with Oct4 plasmids and OAC2, respectively. Proteins in CM were analyzed in the Dionex UltiMate 3000 RSLC nano-system combined with the Q-exactive high-field hybrid quadrupole orbitrap mass spectrometer (Thermo Fisher Scientific). Proteins were first digested on beads using trypsin/LysC as described previously 58, 59 except digestion was performed in 50 mM ammonium bicarbonate buffer instead of urea. Digested peptides were then desalted using mini spin C18 spin columns (Nest Group, Southborough, MA, USA) and separated using a trap and 50-cm analytical columns 58, 60. Raw data were processed using MaxQuant (v1.6.3.3) 61 against the Uniprot mouse protein database at a 1% false discovery rate allowing up to 2 missed cleavages. MS/MS counts were used for relative protein quantitation. Proteins identified with at least 1 unique peptide and 2 MS/MS counts were considered for the final analysis.

The recombinant proteins we employed included Eno1, Pkm, Ppia, Hspa5, Aldoa, Lgals1, Filamin A, Eef2, Tpi1, Pgk1, Pfn1, Plec, Nme2, Eef1a1, Myh9, VCL, Hsp90aa1, Calm1 (MBS2009113, MBS8249600, MBS286137, MBS806904, MBS8248528, MBS2086775, MBS962910, MBS1213669, MBS144173, MBS717266, MBS956765, MBS2031199, MBS145412, MBS2033168, MBS717396, MBS957842, MBS142709, MBS2018713; MyBioSource), actin gamma 1, Actn4, Hspa8 (H00000071-P01, H00000081-P01, NBP1-30278; Novus, Littleton, CO, USA), and Hsp90ab1 (OPCA05157; Aviva System Biology, San Diego, CA, USA). In the MTT assay, 5 µg/mL of each of these recombinant proteins were added and the viability of tumor cells was evaluated.

### Statistical analysis

For cell-based experiments, three or four independent experiments were conducted and data were expressed as mean ± S.D. In animal experiments, the sample size was chosen to achieve a power of 80% with *p* < 0.05. The primary experimental outcome was tumor weight for the mammary fat pad experiment and the bone volume ratio (BV/TV) for the tibia experiment. The secondary experimental outcome was tumor size for the mammary fat pad experiment and the trabecular number (Tb.N) for the tibia experiment. Statistical significance was evaluated using a one-way analysis of variance (ANOVA). Post hoc statistical comparisons with control groups were performed using Bonferroni correction with statistical significance at *p* < 0.05. The single and double asterisks in the figures indicate *p* < 0.05 and *p* < 0.01, respectively.

## Supplementary Material

Supplementary figures and table.

## Abbreviations

ANOVA: analysis of variance
BMD: bone mineral density
BV/TV: bone volume ratio
CM: conditioned medium
DMEM: dulbecco's modified Eagle's medium
EdU: 5-ethynyl-2'-deoxyuridine
Eef2: eukaryotic elongation factor 2
Eno1: enolase 1
H&E: hematoxylin and eosin
Hsp90ab1: heat shock protein 90 alpha class B, member 1
iPS: induced pluripotent stem
iTS cells: induced tumor-suppressing cells
Lrp5: low-density lipoprotein receptor-related protein 5
MMP9: matrix metalloproteinase 9
MSCs: mesenchymal stem cells
Runx2: runt-related transcription factor 2
Tb.N: trabecular number
Tb.Sp: trabecular separation
TGFβ: transforming growth factor beta
VCL: vinculin
