# Supplementary material for: Counterintuitive production of tumor-suppressive secretomes from Oct4- and c-Myc-overexpressing tumor cells and MSCs
Source: Theranostics. 2022 Mar 28;12(7):3084–103. doi: 10.7150/thno.70549 (PMC9065181; doi:10.7150/thno.70549)
Supplement: Supplementary file 1 — Supplementary figures and table. [file thnov12p3084s1.pdf]

Supplementary Information

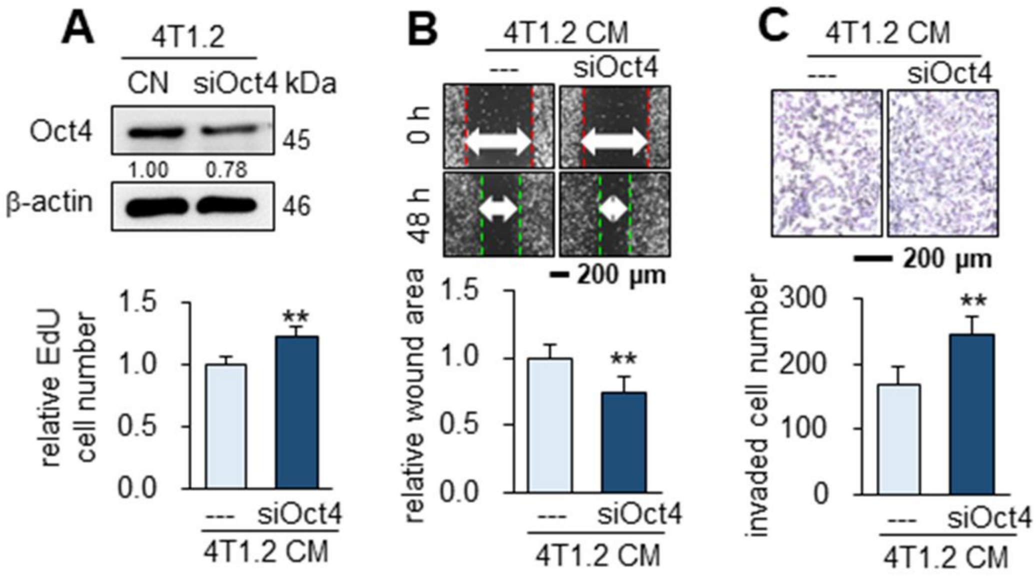

Figure S1. Tumor promotion *in vitro* by Oct4-silencing CM derived from 4T1.2 mammary tumor cells. The double asterisk indicates $p < 0.01$. CN = control, CM = conditioned medium, and siOct4 = Oct4 siRNA. (**A-C**) Elevation in EdU-based proliferation, scratch-based migration, and transwell invasion of parental 4T1.2 cells by Oct4-silenced 4T1.2 cell-derived CM.

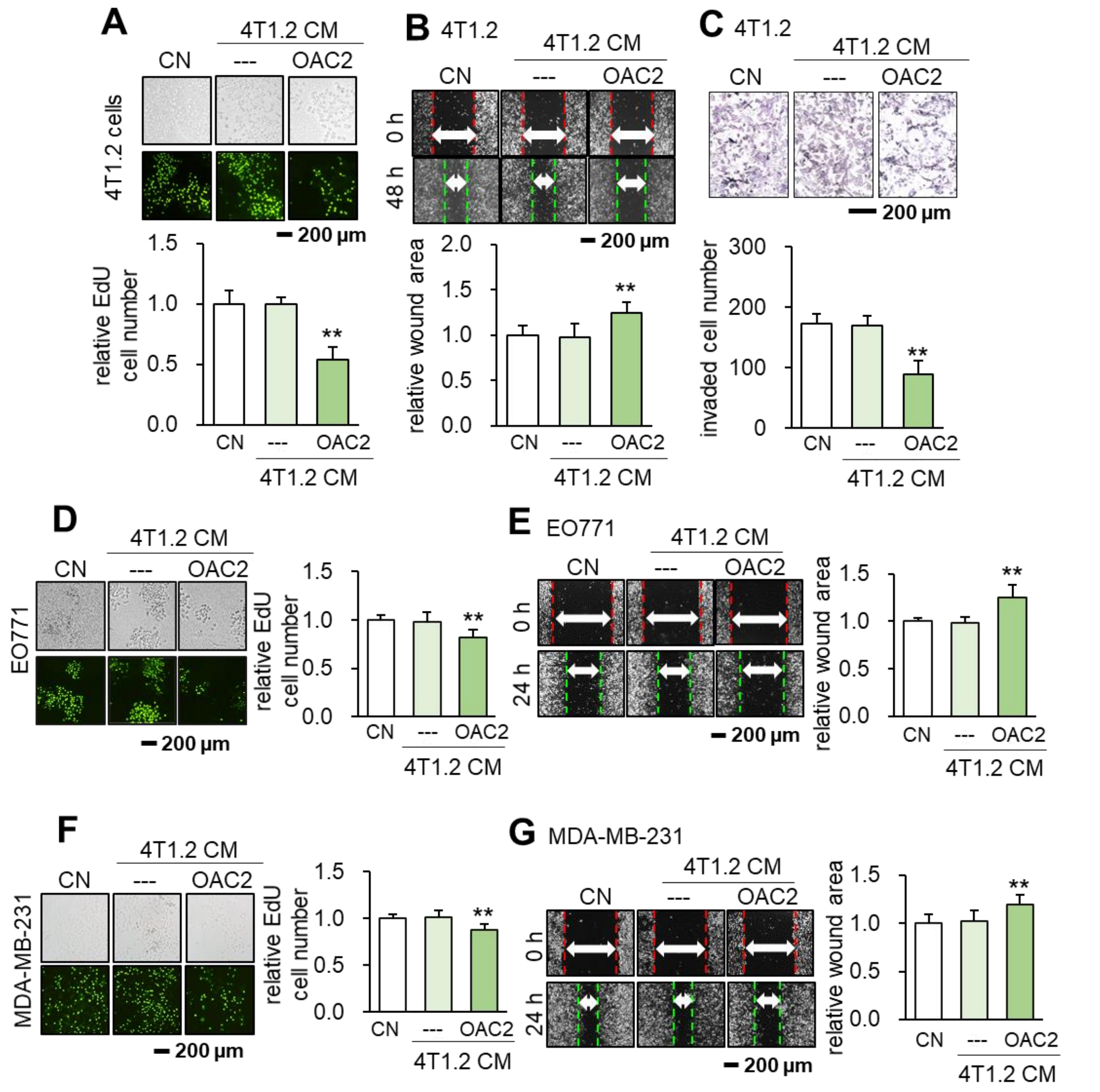

Figure S2. Tumor-suppressing capability of OAC2-treated tumor cell-derived CM. The double asterisk indicates $p < 0.01$. CN = control, and CM = conditioned medium. (**A-C**) Reduction in EdU-based proliferation, scratch-based migration, and transwell invasion in 4T1.2 cells by OAC2 (5 μM)-treated 4T1.2 tumor cell-derived CM. (**D-E**) Reduction in EdU-based proliferation and scratch-based migration in EO771 cells by OAC2-treated 4T1.2 tumor cell-derived CM. (**F-G**) Reduction in EdU-based proliferation and scratch-based migration in MDA-MB-231 breast cancer cells by OAC2-treated 4T1.2 tumor cell-derived CM.

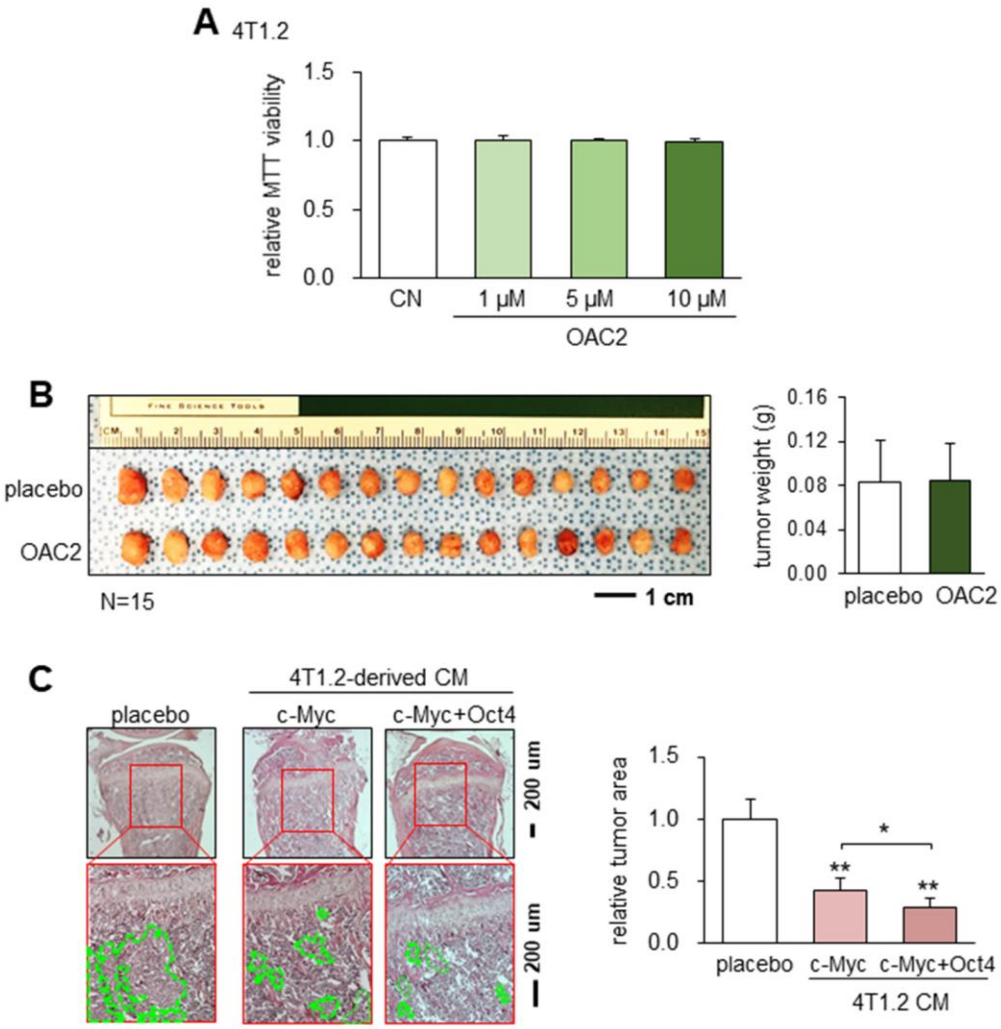

Figure S3. No significant effect by the administration of OAC2 to 4T1.2 tumor cells and mammary tumors, and the effect of c-Myc/Oct4-overexpressing CMs on tumor-driven osteolysis in the tibia. The single and double asterisks indicate $p < 0.05$ and 0.01, respectively. CN = control, CM = conditioned medium, c-Myc = c-Myc plasmids, and c-Myc + Oct4 = c-Myc and Oct4 plasmids. The single and double asterisks indicate $p < 0.05$ and 0.01, respectively. (**A**) No detectable change in MTT-based viability of 4T1.2 cells by the administration of 1 to 10 μM OAC2. (**B**) No detectable change in the size of mammary tumors in BALB/c mice by the daily intraperitoneal injection of 10 mg/kg OAC2. (**C**)Reduction in the tumor-invaded area in the proximal tibia by CM derived from 4T1.2 cells, which was overexpressed with c-Myc and/or Oct4.

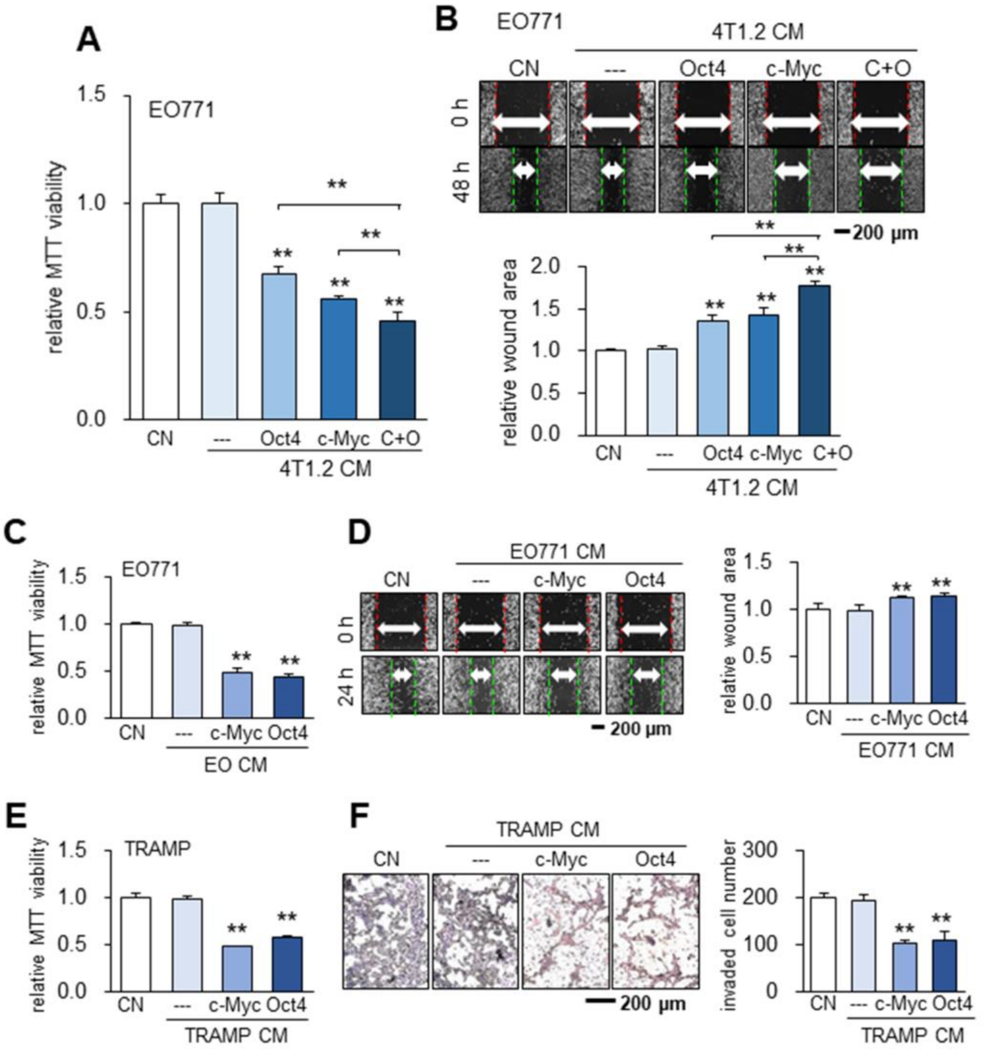

Figure S4. Tumor-suppressing capability of Oct4- and c-Myc-overexpressing 4T1.2 tumor cell-derived CMs in EO771 mammary tumor cells. The double asterisk indicates $p < 0.01$. CN = control, Oct4 = Oct4 plasmids, c-Myc = c-Myc plasmids, C+O = c-Myc and Oct4, and CM = conditioned medium. (**A-B**) Reduction in MTT-based viability and scratch-based migration of EO771 cells by Oct4- and c-Myc-overexpressing 4T1.2 tumor cell-derived CMs. (**C-D**) Inhibition MTT-based viability and scratch-based migration of EO771 mammary tumor cells by c-Myc/Oct4-overexpressing EO771 tumor cell-derived CM. (**E-F**) Inhibition of MTT-based viability and transwell invasion of TRAMP prostate tumor cells by c-Myc/Oct4-overexpressing TRAMP tumor cell-derived CM.

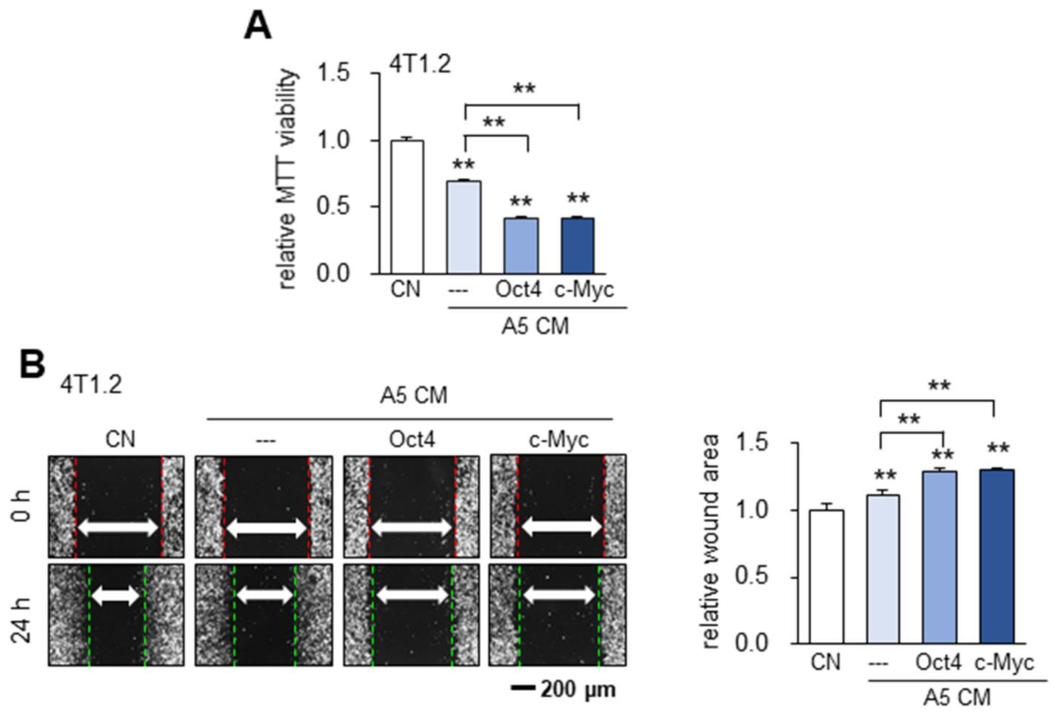

Figure S5. Tumor-suppressing capability of Oct4- and c-Myc-overexpressing non-tumor cell-derived CMs in 4T1.2 mammary tumor cells. The double asterisk indicates $p < 0.01$. CN = control, A5 = MLO-A5 osteocytes, Oct4 = Oct4 plasmids, c-Myc = c-Myc plasmids, and CM = conditioned medium. (**A-B**) Reduction in MTT-based viability and scratch-based migration of 4T1.2 cells by Oct4- and c-Myc-overexpressing MLO-A5 osteocytes-derived CM.

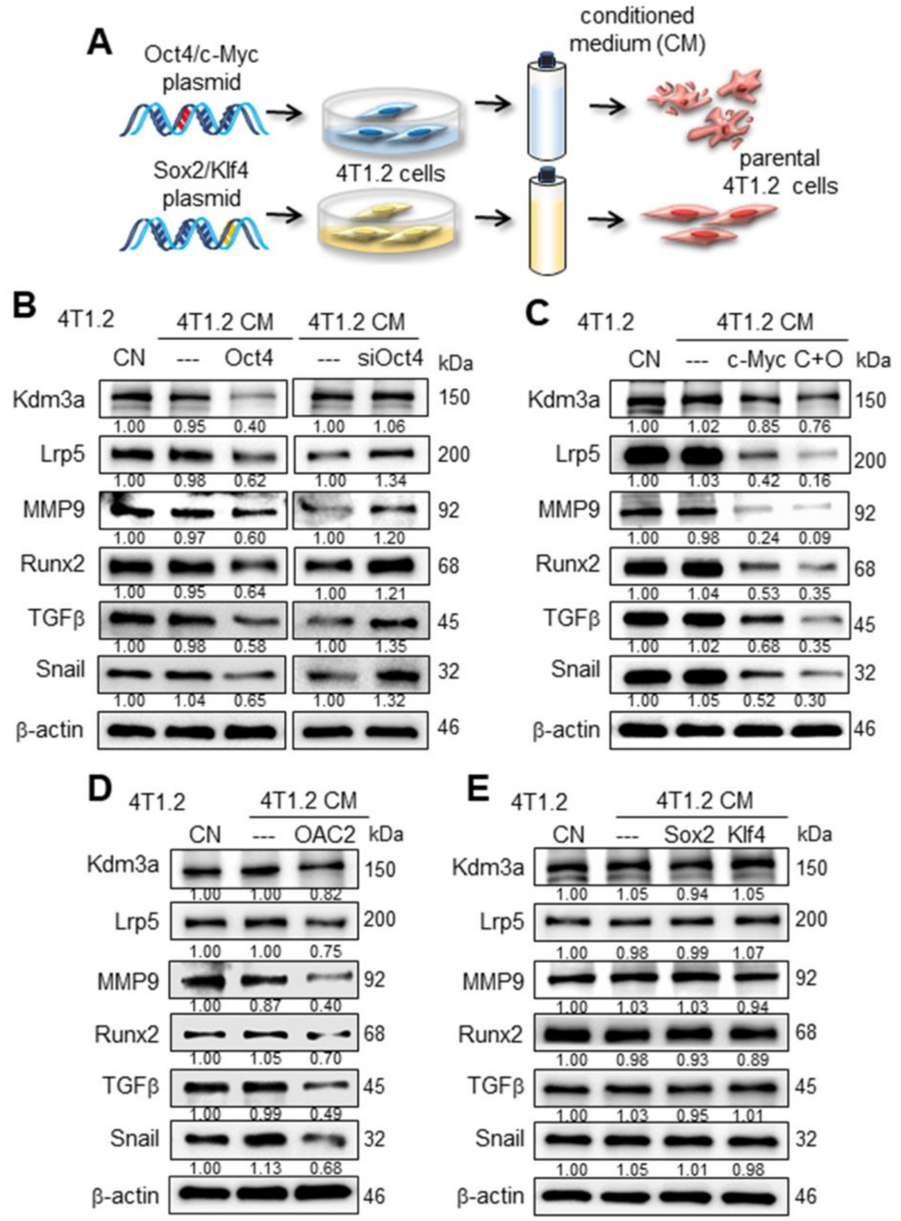

Figure S6. Effects of the Yamanaka factors (Oct4, c-Myc, Sox2, and Klf4) on Kdm3a and the selected tumor-promoting genes (Lrp5, MMP9, Runx2, TGFβ, and Snail). CN = control, CM = conditioned medium, Oct4 = Oct4 plasmids, siOct4 = Oct4 siRNA, c-Myc = c-Myc plasmids, C+O = c-Myc and Oct4, Sox2 = Sox2 plasmids, and Klf4 = Klf4 plasmids. (**A**) Plasmid-based overexpression and siRNA-based silencing of Yamanaka factors to generate tumor cell-derived CM. (**B**) Downregulation of Kdm3a and the tumor-promoting genes by Oct4 overexpression and their upregulation by Oct4 silencing in 4T1.2 cells. (**C-D**) Downregulation of Kdm3a and the tumor-promoting genes by c-Myc and Oct4 overexpression, as well as OAC2 treatment in 4T1.2 cells. (**E**) No detectable change in the expression of Kdm3a and the selected tumor-promoting genes by the overexpression of Sox2 and Klf4 in 4T1.2 cells.

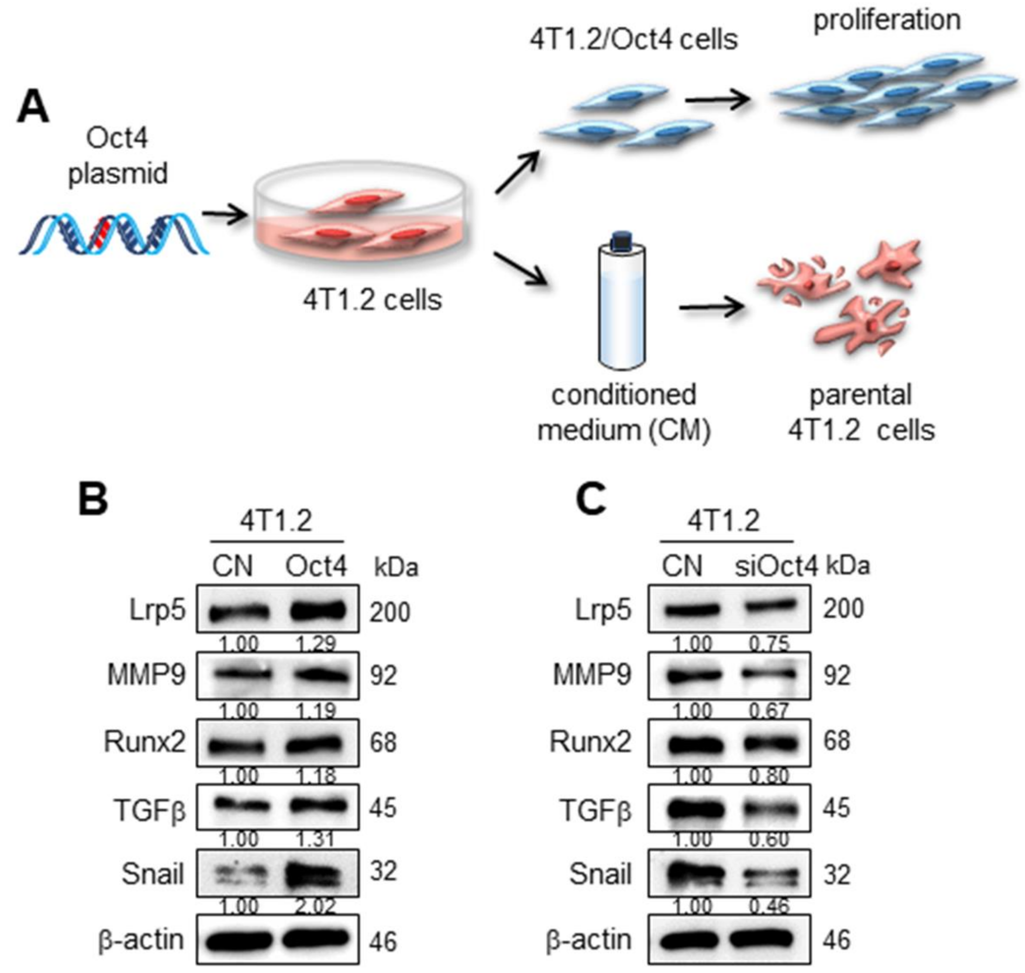

Figure S7. Stimulatory effects of the overexpression and silencing of Oct4 in 4T1.2 mammary tumor cells for their oncogenic actions. CN = control, Oct4 = Oct4 plasmids, and siOct4 = Oct4 siRNA. (**A**) Overexpression and silencing of Oct4 in 4T1.2 cells by plasmid transfection and RNA interference. (**B**) Elevation in the levels of Lrp5, MMP9, Runx2, TGFβ, and Snail by the overexpression of Oct4 in 4T1.2 cells. (**C**) Reduction in the levels of Lrp5, MMP9, Runx2, TGFβ, and Snail by the silencing of Oct4 in 4T1.2 cells.

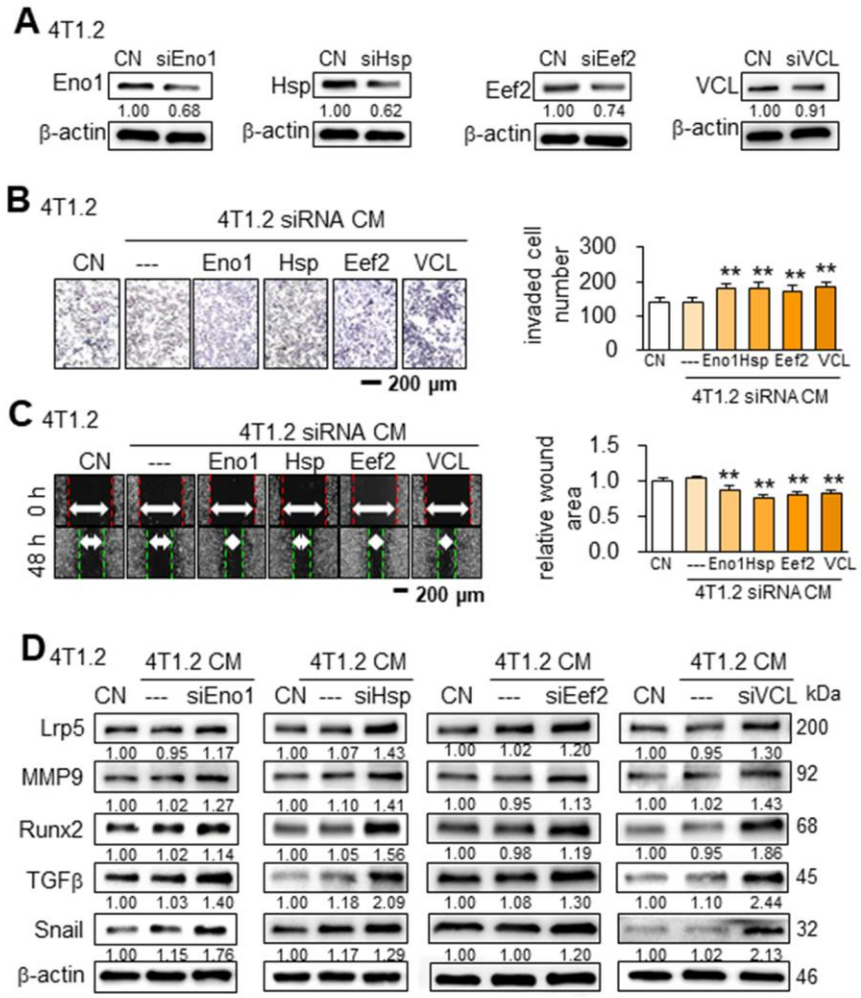

Figure S8. Effects of silencing enolase 1 (Eno1), Hsp90ab1 (Hsp), Eef2, and vinculin (VCL) in 4T1.2 cells. CN = control, CM = conditioned medium, and si = siRNA. (**A-B**) Elevation of transwell invasion of 4T1.2 tumor cells by CM, which was derived from 4T1.2 cells treated with siRNAs specific for Eno1, Hsp90ab1, Eef2, and VCL. (**C**) Stimulation of scratch-based migration of 4T1.2 tumor cells by 4T1.2 cell-derived CM, which was treated with siRNAs specific to Eno1, Hsp90ab1, Eef2, and VCL.(**D**) Elevation of the levels of Lrp5, MMP9, Runx2, TGFβ, and Snail in 4T1.2 cells in response to 4T1.2 cell-derived CM, treated with enolase 1 siRNA, Hsp90ab1 siRNA, Eef2 siRNA, and vinculin siRNA, respectively.

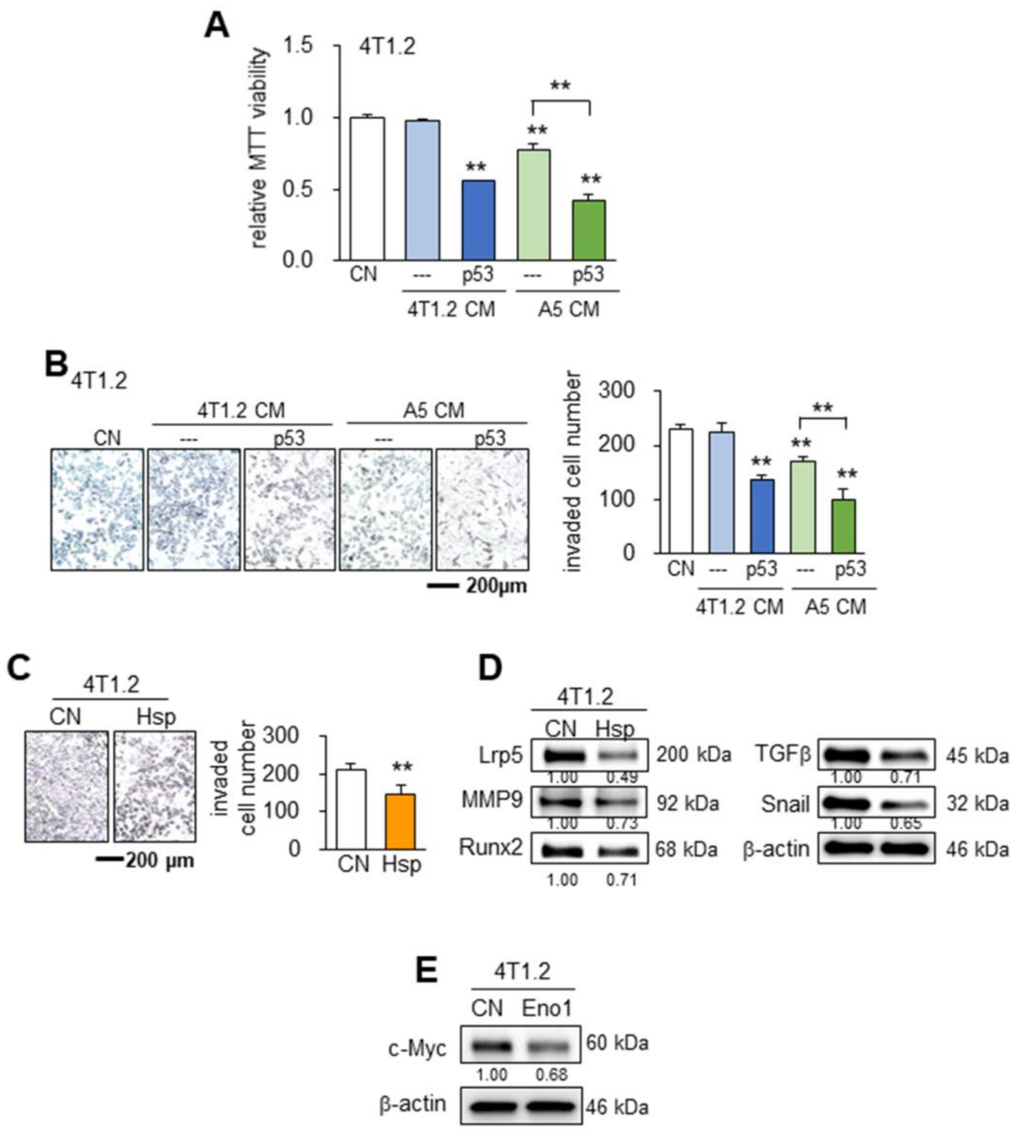

Figure S9. Tumor-suppressing capability of p53-overexpressing tumor and non-tumor cell-derived CMs in 4T1.2 mammary tumor cells, and the effects of Hsp90ab1. The double asterisk indicates $p < 0.01$. CN = control, A5 = MLO-A5 osteocytes, p53 = p53 plasmids, and CM = conditioned medium. (**A-B**) Reduction in MTT-based viability and transwell invasion of 4T1.2 cells by p53-overexpressing 4T1.2 and MLO-A5 osteocyte-derived CM. (**C**) Reduction in transwell invasion by Hsp90ab1 recombinant protein. (**D**) Reduction in the levels of Lrp5, MMP9, Runx2, TGFβ, and Snail by Hsp90ab1 recombinant protein. (**E**)Reduction in c-Myc in 4T1.2 cells by Eno1 recombinant proteins.

Suppl. Table 1. List of 100 proteins that were expressed higher in Oct4-overexpressing and OAC2-treated CMs than the control CM in mass spectrometry-based proteomics analysis.

Num	Gene names	Mol[kDa]	CN	Oct4	OAC2	Num	Gene names	Mol[kDa]	CN	Oct4	OAC2
1	Hspa8	70.9	74	328	250	51	Tpm3;Tpm3-rs7	29.0	20	38	37
2	Eno1	47.1	61	296	236	52	Iqgap1	188.8	0	16	18
3	Pkm	57.8	51	192	166	53	Prdx1	22.2	7	26	22
4	Ppia	18.0	44	180	128	54	Fkbp4	51.6	3	24	15
5	Hspa5	72.4	46	122	158	55	Lgals3	19.9	14	39	22
6	Hsp90ab1	83.3	37	121	120	56	Mdh2	35.6	0	13	19
7	Aldoa	39.4	36	123	105	57	Sptan1	282.9	0	14	18
8	Lgals1	14.9	19	99	87	58	Hnrnpa2b1	37.4	14	31	29
9	Flna	280.5	31	102	91	59	Cmpk2	50.0	0	26	5
10	Eef2	95.3	13	80	62	60	Ak2	26.5	4	22	16
11	Tpi1	32.2	12	72	55	61	Cct8;Cctq	59.6	0	20	10
12	Actn4	105.0	9	63	54	62	Idh1	46.7	2	15	18
13	Vcp	89.3	9	66	47	63	Cct3	60.6	2	18	15
14	Actg1	41.8	32	70	88	64	Uba1	117.8	0	16	13
15	Pgk1	44.6	8	59	48	65	Gdi2	50.5	0	17	12
16	Flnb	277.8	5	61	38	66	Rplp2	11.7	2	23	10
17	Pfn1	14.9	9	68	39	67	Hist1h2bj;Hist1h2bk	13.6	11	25	25
18	Plec	506.5	13	63	46	68	Psma7;Psma8	27.9	2	16	16
19	Ldha	36.5	13	63	46	69	Mtap	31.1	4	20	15
20	Nme2	30.2	17	67	43	70	Wdr1	66.4	2	19	12
21	Eef1a1	50.1	29	73	59	71	G3bp1	56.2	0	17	10
22	Myh9	226.4	13	36	52	72	Hist1h2ah;Hist1h2aa	13.7	8	20	22
23	Vcl	116.7	10	43	39	73	Grn	63.5	0	12	14
24	Hsp90aa1	84.8	9	42	36	74	Srsf1	28.3	0	13	13
25	Calm1	16.8	18	51	44	75	Pgd	53.3	2	18	12
26	Cfl1	18.6	13	47	38	76	Psme1	27.4	0	17	9
27	GAPDH;Gapdh;m3839	35.8	17	52	40	77	Lap3	56.1	0	18	8
28	Lmna	74.2	30	67	50	78	Atic	64.2	3	14	17
29	Pdia3	56.7	15	47	39	79	Eef1g	50.1	6	22	15
30	Gpi;Gpi1	62.8	2	32	25	80	Ahcy	47.7	0	16	9
31	Msn	67.7	19	50	41	81	Cltc;mKIAA0034	192.0	0	11	13
32	Pgam1	28.8	6	36	28	82	Anxa2	38.6	6	18	18
33	Arhgdia	23.4	11	38	35	83	Serpinb6a;Serpinb6	42.6	0	13	11
34	Fasn	272.4	3	27	29	84	Pnp;Pnp2	32.3	0	21	3
35	Tln1	272.1	0	27	23	85	Psat1	40.5	2	15	12
36	Tuba1b;Tuba1c;Tuba1a	50.2	16	35	46	86	Pls3	70.6	0	13	10
37	Tkt	67.6	16	44	37	87	Ctsl	37.6	42	44	62
38	Ywhae	29.2	11	39	31	88	Ncl	76.9	15	24	28
39	Tagln2	22.4	7	37	24	89	Ctsb	37.3	3	16	12
40	Dpysl2	62.3	8	34	27	90	Pa2g4	43.7	2	15	11
41	Prdx6	24.9	9	40	23	91	Ckb	42.7	0	13	9
42	Flnc	291.1	0	25	16	92	Got1	46.2	0	14	8
43	Gm1821;Ubc;Uba52;Ubb	17.2	23	48	38	93	Cct5	59.6	0	14	8
44	Eif5a;Eif5a2	16.3	6	35	17	94	Sptbn1	274.2	0	10	11
45	Actn1	103.1	3	24	21	95	Mif	12.5	7	18	17
46	Hspa4	94.2	11	35	26	96	Gstp1;Gstp2	23.6	2	14	11
47	Ywhaz	27.8	12	34	28	97	Eprs	170.0	0	12	9
48	Pabpc1	70.7	2	27	15	98	Hmga1	11.6	2	15	10
49	Stip1	62.5	10	37	21	99	Cct2	57.5	0	14	7
50	Tubb5	49.7	12	22	37	100	Vars	140.2	2	17	8

Of note, MS/MS counts were used for the relative protein quantitation. The proteins identified with at least 1 unique peptide and 2 MS/MS counts were considered for the final analysis.
